# Sialylation converts arthritogenic IgG into inhibitors of collagen-induced arthritis

**DOI:** 10.1038/ncomms11205

**Published:** 2016-04-05

**Authors:** Yuhsuke Ohmi, Wataru Ise, Akira Harazono, Daisuke Takakura, Hidehiro Fukuyama, Yoshihiro Baba, Masashi Narazaki, Hirofumi Shoda, Nobunori Takahashi, Yuki Ohkawa, Shuting Ji, Fumihiro Sugiyama, Keishi Fujio, Atsushi Kumanogoh, Kazuhiko Yamamoto, Nana Kawasaki, Tomohiro Kurosaki, Yoshimasa Takahashi, Koichi Furukawa

**Affiliations:** 1 Department of Biochemistry II, Nagoya University Graduate School of Medicine, 65 Tsurumai, Showa-ku, Nagoya 466-0065, Japan; 2 Department of Immunology, National Institute of Infectious Diseases, 1-23-1 Toyama, Shinjuku-ku, Tokyo 162-8640, Japan; 3 Laboratory of Lymphocyte Differentiation, WPI Immunology Frontier Research Center and Graduate School of Frontier Biosciences, Osaka University, 3-1 Yamadaoka, Suita, Osaka 565-0871, Japan; 4 Division of Biological Chemistry and Biologicals, National Institute of Health Sciences, 1-18-1 Kamiyoga, Setagaya-ku, Tokyo 158-8501, Japan; 5 Laboratory of Proteome Science, Graduate School of Medical Life Science, Yokohama City University, 1-7-29, Suehiro, Tsurumi-ku, Yokohama, Kanagawa 230-0045, Japan; 6 Laboratory for Lymphocyte Differentiation, RIKEN Center for Integrative Medical Sciences, 1-7-22, Suehirocho, Tsurumi-ku, Yokohama, Kanagawa 230-0045, Japan; 7 Department of Respiratory Medicine, Allergy and Rheumatic Diseases, Osaka University Graduate School of Medicine, 2-2 Yamadaoka, Suita, Osaka 565-0871, Japan; 8 Department of Allergy and Rheumatology, Graduate School of Medicine, The University of Tokyo, 7-3-1 Hongo, Bunkyo-ku, Tokyo 113-0033, Japan; 9 Department of Orthopedics, Nagoya University Graduate School of Medicine, 65 Tsurumai, Showa-ku, Nagoya 466-0065, Japan; 10 Department of Biomedical Sciences, Chubu University College of Life and Health Sciences, 1200 Matsumoto, Kasugai 487-8501, Japan; 11 Laboratory Animal Resource Center, University of Tsukuba, 1-1-1 Ten-no-dai, Tsukuba 305-8575, Japan

## Abstract

Rheumatoid arthritis (RA)-associated IgG antibodies such as anti-citrullinated protein antibodies (ACPAs) have diverse glycosylation variants; however, key sugar chains modulating the arthritogenic activity of IgG remain to be clarified. Here, we show that reduced sialylation is a common feature of RA-associated IgG in humans and in mouse models of arthritis. Genetically blocking sialylation in activated B cells results in exacerbation of joint inflammation in a collagen-induced arthritis (CIA) model. On the other hand, artificial sialylation of anti-type II collagen antibodies, including ACPAs, not only attenuates arthritogenic activity, but also suppresses the development of CIA in the antibody-infused mice, whereas sialylation of other IgG does not prevent CIA. Thus, our data demonstrate that sialylation levels control the arthritogenicity of RA-associated IgG, presenting a potential target for antigen-specific immunotherapy.

## Abstract (web summary)

Post-translational modifications, such as glycosylation and sialylation, are thought to confer disease modifying effects on autoimmune-associated antibodies, including anti-citrullinated protein antibodies in rheumatoid arthritis. Here the authors show that sialylation converts arthritogenic IgG into inhibitors of collagen-induced arthritis in mice.

## Graphical abstract

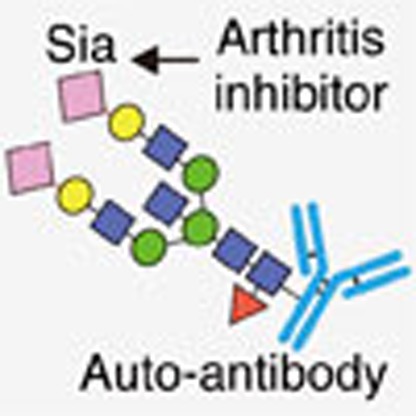

Rheumatoid arthritis (RA) is a chronic autoimmune disorder characterized by joint destruction and inflammation. Although the combination of biologic and conventional synthetic disease-modifying anti-rheumatic drugs has improved the quality of life for patients with RA, profound remission is only achieved by 5–10% of treated patients^1^. Moreover, because current treatments are based on antigen-non-specific suppression of immune responses, concomitant suppression of protective immunity to infectious pathogens may increase the risk of adverse effects. Therefore, new immunomodulatory therapies are needed to selectively target RA-associated autoimmune reactions.

Development of antigen-specific therapy for RA has been hampered by insufficient knowledge of the autoimmune reactions underlying disease pathogenesis. Previous studies have identified autoantigens and autoantibodies that are potentially relevant to RA pathogenesis. Among them, anti-citrullinated protein antibodies (ACPAs) are highly specific to RA patients and are applied as the most specific biomarker for diagnosis and prognosis prediction^2^,^3^. Moreover, several clinical observations indicate the pathogenic potential of ACPAs: the presence of synovial ACPAs precedes the clinical manifestations of arthritis^4^,^5^,^6^, and ACPA^+^ immune complexes promote proinflammatory cytokine secretion by macrophages^7^. Furthermore, the osteoclastogenic ability of ACPAs and the immune complexes can contribute to RA pathogenesis^8^,^9^. Indeed, the arthritogenic activity of ACPAs was demonstrated in a mouse model of RA in which passive transfer of mouse ACPAs induced inflammatory arthritis with a clinical signature comparable to that of human RA^10^,^11^, although such arthritogenic activity is observed not only in ACPAs, but also in collagen-binding antibodies. Thus, further understanding of autoantibody-mediated RA pathogenesis would accelerate the development of novel antigen-specific immunotherapies for RA-associated joint destruction and inflammation.

Autoantibodies including ACPAs are typically of the IgG isotype, which carry one conserved *N*-glycosylation site at Asn297 on each of their Fc regions^12^. Intriguingly, patients with progressive RA have poorly galactosylated and sialylated IgG compared with patients with less severe disease or those in remission^13^,^14^,^15^,^16^. Degalactosylated IgG enhance pathogenic activity in several autoimmune disease models^17^,^18^. Conversely, galactosylated IgG counteract complement-mediated inflammation^19^, supporting the concept that galactosylation levels influence the pathogenic potential of IgG. Likewise, sialylation of IgG Fc confers regulatory function on disease-associated antibodies, whereas non-sialylated IgG exaggerates antigen-dependent inflammation^20^,^21^. Also, Ravetch *et al.* reported that the sialylated fraction of intravenous immunoglobulin G (IVIG) is effective in its anti-inflammatory activity^22^, although sialylation-independent effects have also been observed^23^,^24^. Therefore, modulation of glycosylation on disease-associated IgG might be vital to develop an immunomodulatory therapy that selectively targets disease-associated autoimmune reactions.

Here we show that the sialylation process contributes to disease pathogenesis in the collagen-induced arthritis (CIA) mouse model, a model that mirrors many clinical and immunological features of RA. Enforced sialylation of mouse collagen antibodies, including ACPAs, reverses proinflammatory activity and provides a regulatory function in CIA, whereas the sialylation of other IgG was not preventive of disease. Thus, the sialylation of IgG Fc converts ACPAs from a ‘pathogenic' form to a ‘regulatory' form, providing a feasible approach to control RA pathogenesis.

## Results

### IgG Fc is desialylated in RA patients

**Figure 1:**
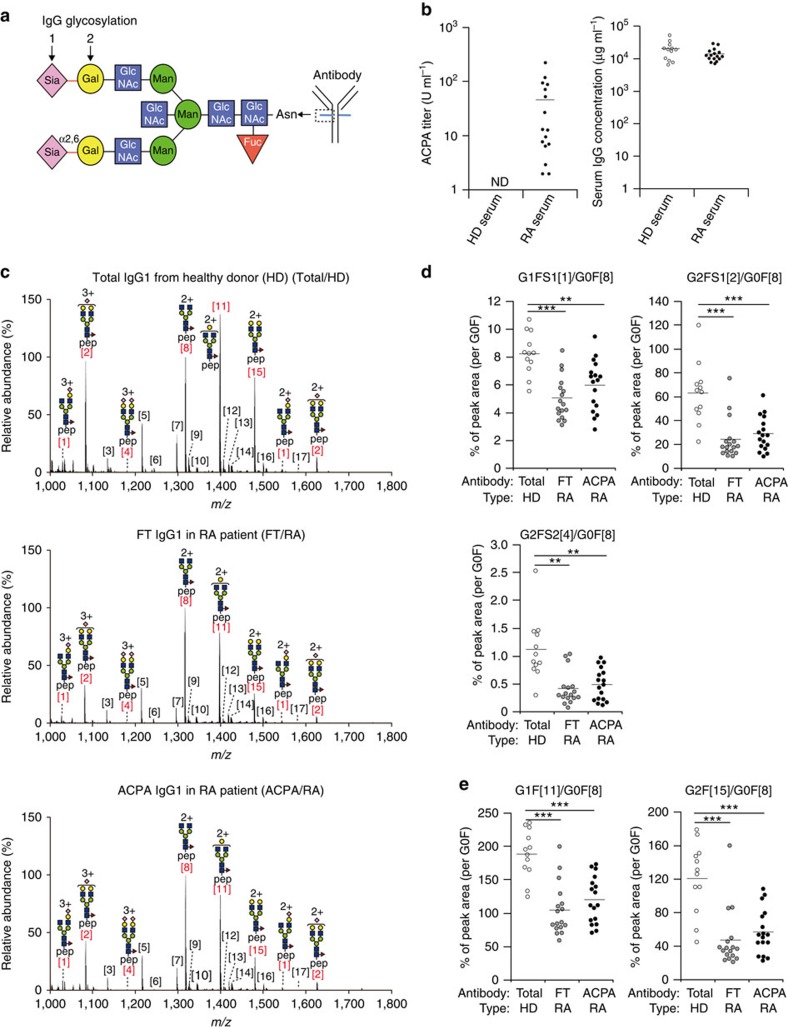
IgG Fc in RA patients is desialylated because of lack of galactosylation. (**a**) Structure of *N*-glycosylation forms attached to Asn297 in the IgG Fc portion. Sugar chains of IgG comprise bi-antennary chains, and are further modified by binding bisecting *N*-acetylglucosamine (GlcNAc), core-fucosylation and terminal α2,6-linkage of sialic acid to galactose. Sia, sialic acid; Gal, galactose; Man, mannose; Fuc, fucose. (**b**) CCP-binding IgG (ACPA) titres and total IgG levels from RA patients (*n*=17) or healthy donors (*n*=12) are presented. Each symbol represents the data from an individual donor. Mean values were presented as bars. ND, not detectable. (**c**) LC-ESI-MS analysis of IgG1 Fc glycans in total IgG from HDs (Total/HD), and those in the flow-through (FT) of CCP columns (FT/RA) and CCP-binding IgG ACPA (ACPA/RA) from RA patients. Glycoforms of human IgG1 Fc are shown in Supplementary Table 1. pep, peptide moiety (EEQYNSTYR). (**d**,**e**) Ratios of sialylated (**d**) and galactosylated (**e**) IgG Fc glycans to agalactosylated IgG Fc glycan (G0F) in each group of serum were calculated and plotted. Each circle represents the result from an individual donor. Mean values were presented as bars. Data were analysed by Steel–Dwass non-parametric test (**P<0.01; ***P<0.001).

Human and murine IgG of all subclasses have a conserved *N*-glycosylation site at Asn297 on their Fc regions (Fig. 1a). *N*-glycans on IgG Fc have been classified into three subgroups: the first ending with *N*-acetylglucosamine (GlcNAc) (G0F), the second with galactose on one arm (G1F) and the third with two galactose on both arms (G2F) (Fig. 1a). A minor fraction of galactosylated glycan can be further sialylated with either one (G1FS1 or G2FS1) or two sialic acid residues (G2FS2) (Fig. 1a). Agalactosylated glycoforms (G0F) are frequently observed in serum IgG of RA patients^13^,^16^. Likewise, agalactosylated glycoforms are observed in murine IgG in autoimmune-prone mouse strains such as the MRL/lpr strain^25^,^26^; however, the glycosylation profiles of RA-associated IgG have not been directly compared in RA patients and mouse models. We assessed RA-associated glycosylation profiles on IgG ACPA and total IgG in ACPA^+^ RA patients. ACPA^+^ RA patients showed high amounts of cyclic citrullinated peptide (CCP)-binding IgG (ACPA) but normal total IgG levels in their serum (Fig. 1b). Three types of IgG fractions were purified and subjected to liquid chromatography-electrospray ionization-mass spectrometry (LC-ESI-MSI) analysis: serum ACPA purified by the binding ability to CCP from ACPA^+^ RA patients (ACPA/RA), total serum IgG deprived of ACPA from the same RA patients (FT/RA), and total serum IgG from healthy donors (total/HD) (Fig. 1c; Supplementary Fig. 1). To assess the antigen specificity of purified ACPA, the binding of purified ACPA to CCP and arginine-control peptide was compared in parallel (Supplementary Fig. 1). We observed that the binding of purified ACPA to the control peptide was 100-fold lower than that to CCP. Moreover, a competitive ELISA (enzyme-linked immunosorbent assay) confirmed the lack of binding to the control peptide because CCP binding of the purified ACPA was blocked by the addition of CCP but not by control peptide. However, low recovery of antibodies from ACPA-negative RA patients suggests that small amounts of non-ACPA is included in ACPA fraction through the purification process as previously noted^27^ (Supplementary Fig. 2).

Consistent with previous reports^16^, the frequencies of terminally sialylated glycoforms, G1FS1 (peak 1), G2FS1 (peak 2), and G2FS2 (peak 4) were equivalently reduced in both IgG1 ACPA and total IgG1 from RA patients compared with total IgG1 from healthy donors; no difference was found between ACPA and total IgG1 from RA patients (Fig. 1d). As sialic acid is covalently attached to galactose, reduced sialylation may represent either a lack of sialic acid itself (Fig. 1a, arrow 1) or a lack of galactosylation (Fig. 1a, arrow 2). To discriminate between these possibilities, we analysed the ratios of galactosylated G1F (peak 11) and G2F (peak 15) glycoforms per G0F backbone (peak 8). The frequency of galactosylation was reduced in both ACPA and total IgG1 in RA patients to levels comparable to those with terminal sialylation (Fig. 1e). In fact, galactose-containing glycoforms were equivalently sialylated in all three types of IgG1 (Supplementary Fig. 3). Therefore, these results indicate that the reduced sialylation of Fc primarily reflects a lack of galactose rather than defective terminal sialylation. Glycosylation profiles of IgG2 subclass were consistent with those observed for IgG1, indicating that this is a common event in all IgG classes (Supplementary Fig. 4). It is unlikely that the contaminating non-ACPA antibodies affect the results, because the removal of non-specific antibodies using an arginine-control-peptide-coated column did not demonstrate any difference in the glycan profiles (Supplementary Fig. 5).

### IgG Fc is desialylated in arthritis mouse models

**Figure 2:**
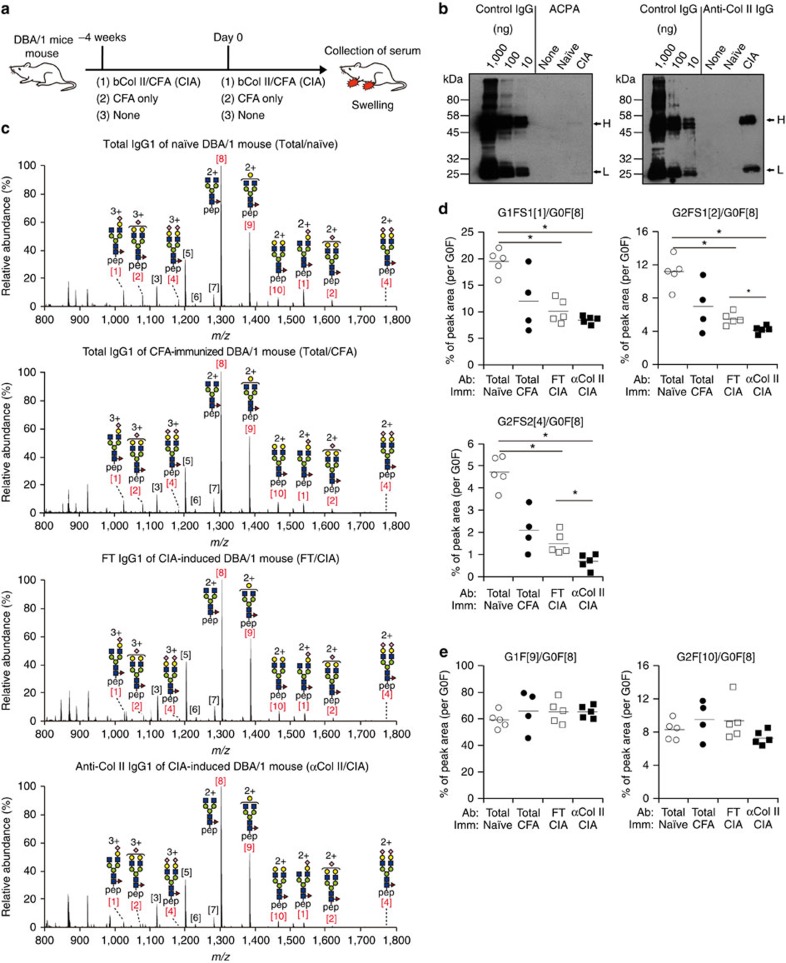
Sialylation, but not galactosylation, levels on IgG Fc decreased in CIA mice. (**a**) An experimental scheme for collagen-induced arthritis (CIA). DBA/1 mice were primed with either bovine Col II (bCol II) in complete Freund's adjuvant (CFA) or CFA alone on −4 weeks. At day 0, DBA/1 mice were boosted with the same antigens and sera were collected at day 14. (**b**) CCP2-binding ACPA and anti-Col II antibodies were collected from 50 μl (ACPA) and 1 μl (Col II) of the serum from naive and CIA mice, and then subjected to western blotting using purified mouse IgG (10, 100 and 1,000 ng per each lane) as reference. (**c**) LS-ESI-MS analysis of IgG1 Fc glycans in the total IgG from naive (total/naive) or CFA-immunized mice (total/CFA) and those in the flow-through of Col II column (FT/CIA) or anti-Col II IgG1 (αCol II/CIA) from CIA-induced mice. Glycoforms of DBA/1 mouse IgG1 Fc are shown in Supplementary Table 2. (**d**,**e**) Ratios of sialylated (**d**) or galactosylated (**e**) IgG Fc glycans to the agalactosylated IgG Fc glycan (G0F) in the indicated groups were calculated and plotted. Each circle represents the result from an individual mouse. Mean values were presented as bars. Data were analysed by Steel–Dwass non-parametric test (*P<0.05).

The glycosylation profiles of antigen-specific and total IgG1 were assessed in a CIA mouse model after immunization with adjuvanted type II collagens (Col II). We used highly susceptible DBA/1 strains, all of which developed joint inflammation after booster immunization (Fig. 2a). Although the CIA mouse model is widely used to mimic the antibody-dependent process of RA pathogenesis, conflicting reports on ACPA induction in this mouse model exist^10^,^28^. Indeed, at least in the experimental conditions defined by us, ACPAs were produced at insufficient levels for glycosylation profiling by mass spectrometry (Fig. 2b). Hence, anti-Col II IgG antibodies, which have arthritogenic activity^29^,^30^,^31^, were purified for glycosylation profiling (Supplementary Fig. 6). We prepared four types of serum IgG fractions for LC-ESI-MS analysis: anti-Col II antibody from CIA mice (αCol II/CIA), total IgG deprived of anti-Col II antibody from the same mice (FT/CIA) and total serum IgG from CFA primed (total/CFA) or naive mice (total/naive) (Fig. 2c; Supplementary Fig. 6b,c). The ratios of sialylated glycoforms were significantly reduced in anti-Col II and total IgG1 of CIA mice relative to naive mice; the reduction was significantly exaggerated in bi-galactosylated forms of anti-Col II IgG1 (Fig. 2d). Sialylation was also reduced in total IgG1 from CFA-injected mice, albeit at an insignificant level. The glycoforms containing terminal galactose residues were comparable among the four groups in non-CIA and CIA mice (Fig. 2e) and similar results were obtained for IgG2a/b subclasses (Supplementary Fig. 7). Thus, these results indicate that poor sialylation of IgG Fc is a feature common to both RA patients and CIA mice, although the underlying mechanisms may not be identical.

Because CIA mouse model do not completely replicate RA in humans, we decided to analyse another RA model mice which express human HLA-DR4 known to be associated with RA^32^,^33^. Immunization of this mouse strain with citrullinated fibrinogen (cFib) induces joint inflammation concomitant with the induction of cFib-specific B- and T-cell responses, mimicking clinical and immunological features of RA patients (Supplementary Fig. 8a). However, similar to that observed with the previous CIA model, ACPAs were again produced at insufficient levels for glycosylation profiling. Therefore, following RA induction, we purified three types of serum IgG fractions for LC-ESI-MS analysis: anti-Fib antibody from cFib-immunized mice (Fib/cFib), total IgG deprived of anti-Fib antibody in the same mice (FT/cFib) and total IgG from naïve mice (total/naive) (Supplementary Figs 8 and 9). As observed for the CIA model, sialylated IgG1 was significantly reduced in cFib-immunized transgenic mice compared with naive transgenic mice, but the galactosylation level was comparable (Supplementary Figs 9 and 10). Thus, the comparative glycosylation profiling of RA patients and two independent mouse models indicates that desialylation of IgG Fc is associated with RA disease in both species, although the changes in Fc-sialylation are also observed in other inflammatory conditions^34^.

### Loss of sialylation exacerbates joint inflammation in CIA

**Figure 3:**
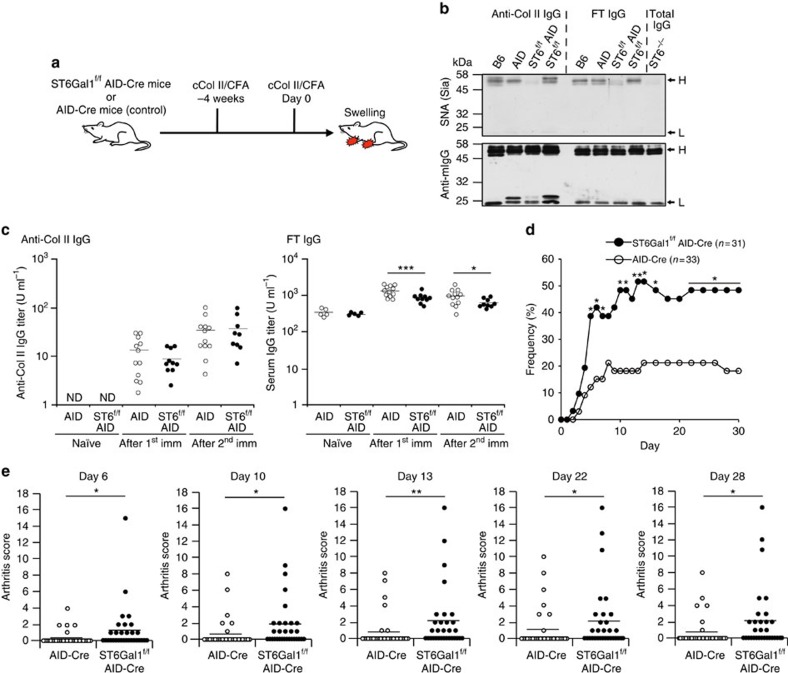
Loss of sialylated IgG Fc by gene targeting exacerbates joint inflammation in CIA model. (**a**) An experimental scheme for CIA induction is presented. Mice of different genotypes were immunized with chicken Col II (cColI II)/CFA at 4-week intervals. Mice were monitored daily for the incidence and scores of arthritis for 14 days. (**b**) Sialylation levels of anti-Col II IgG and FT from the indicated genotypes were assessed by lectin blot analysis. The locations of heavy (H) and light chains (L) are indicated by arrows. (**c**) Anti-Col II IgG and total IgG titres in serum were determined by ELISA after first and second immunization. Each circle represents the result from an individual mouse. Mean values were presented as bars. ND, not detectable. The data are representative of three independent experiments. Data were analysed by two-tailed Student's *t* test (*P<0.05; ***P<0.001). (**d**,**e**) Arthritis frequency (**d**) and swelling score or ankylosis score (**e**) in ST6Gal1^f/f^ AID-Cre (*n*=31) and AID-Cre (*n*=33) mice. Each circle represents the result from an individual mouse. Mean values were presented as bars. The data are representative of two independent experiments. Arthritis frequency (**d**) was analysed by two-tailed Fisher's exact test (*P<0.05; **P<0.01) and swelling scores (**e**) were analysed by Mann–Whitney non-parametric test (*P<0.05; **P<0.01).

The elevated levels of desialylated IgG observed in RA patients and mouse models led us to investigate the direct link between desialylated IgG and the progression of RA pathogenesis. While degalactosylated IgG has been suggested to exacerbate the pathogenesis of RA^17^, the role of desialylated IgG in the RA mouse model has not been addressed. Here, we utilized a gene-targeting approach to block the sialylation of endogenously produced IgG. Sialylation of IgG Fc is carried out by the glycosyltransferases, namely, ST6Gal1 and/or ST6Gal2, that catalyse the transfer of a sialic acid to galactose with α2,6-linkage. The expression level of *mSt6gal2* gene was much lower than *mSt6gal1* in all tissues examined (Supplementary Fig. 11). Therefore, we crossed ST6Gal1^f/f^ with mice expressing Cre recombinase under the control of activation-induced cytidine deaminase gene promoter (AID-Cre) (ref. 35) (Supplementary Fig. 12), which is selectively activated in stimulated B cells. Both homozygous (ST6Gal1^f/f^ AID-Cre^+^) and control AID-Cre (ST6Gal1^+/+^ AID-Cre^+^) mice were immunized with adjuvanted chicken Col II twice to induce CIA (Fig. 3a). To assess the sialylation levels of serum IgG in these mice, we purified both anti-Col II IgG and total IgG from each genotype of mice after CIA induction. Sambucus nigra (SNA) lectin blot and MALDI-TOF-MS analysis revealed the reduced sialylation of total IgG from ST6Gal1^−/−^ and ST6Gal1^f/f^ AID-Cre mice, confirming the major contribution of ST6Gal1 to IgG Fc-sialylation (Fig. 3b; Supplementary Figs 13 and 20). We then analysed anti-Col II IgG titres in CIA mice and observed that homozygous mice produced comparable levels of anti-Col II IgG after priming and boosting of Col II, although total IgG levels were slightly but significantly reduced in homozygous mice (Fig. 3c; Supplementary Fig. 14). Therefore, deficient ST6Gal1 expression in activated B cells did not cause severe defects in B-cell pathways for arthritis-associated IgG production. However, despite equivalent production of arthritis-associated IgG, homozygous mice developed CIA at earlier time points and exhibited a >2-fold higher incidence of CIA compared with control mice (Fig. 3d). More severe joint swellings or ankylosis of the limb were also observed in homozygous mice (Fig. 3e). These data clearly demonstrate that sialylation levels of activated B-cell-intrinsic molecules regulate joint inflammation. Although there are several other candidate molecules (i.e., cytokines) that may be involved in this process, here we focused on sialylation levels of IgG Fc as the reduced sialylation of IgG Fc was common feature in RA patients and mouse models (Figs 1, 2, 3).

### Sialylation diminishes the arthritogenic activity of ACPAs

**Figure 4:**
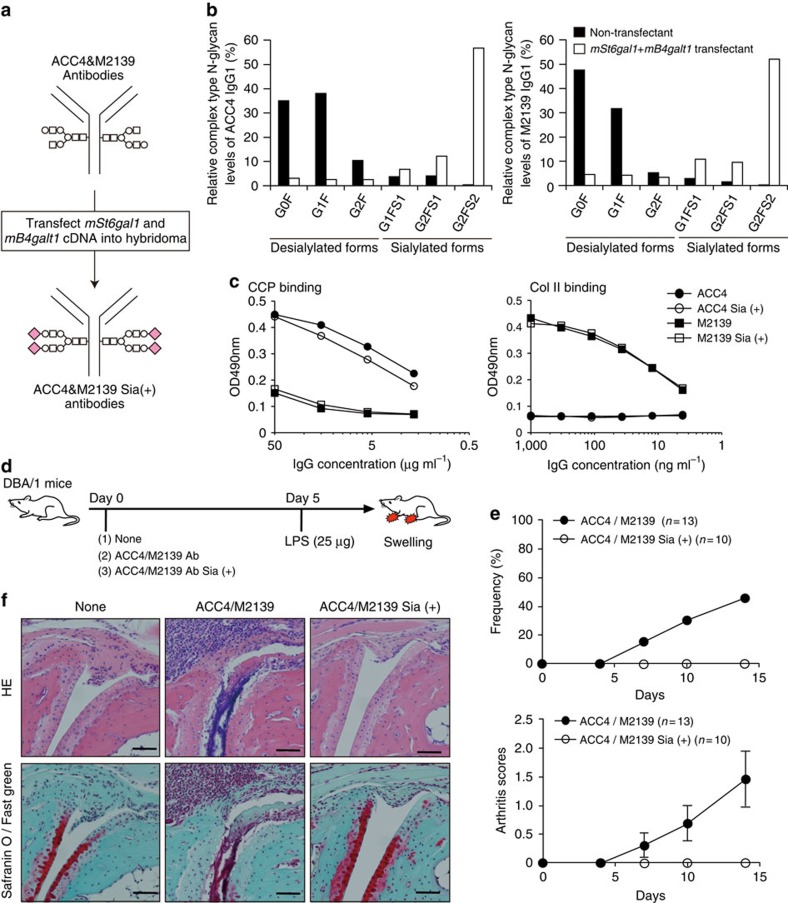
Enforced sialylation reduces the arthritogenic activity of ACPAs. (**a**) An experimental scheme for enforced sialylation. (**b**) LC-ESI-MS analysis of ACC4 and M2139 was performed to detect desialylated glycoforms (G0F, G1F and G2F) and sialylated glycoforms (G1FS1, G2FS1 and G2FS2) of IgG Fc glycans. To normalize the variability, summation of peak areas of all complex type *N*-glycans were deliberately set at 100%. (**c**) Antigen-binding ability of Sia (+) and control ACC4/M2139 was compared by ELISA. (**d**) An experimental scheme for CAIA. (**e**) Frequency (upper panel) and score (lower panel) of arthritis is plotted. Data for arthritis scores are shown as mean±s.d. (*n*=13 for ACC4/M2139 and *n*=10 for ACC4/M2139 Sia (+)). The data are representative of two independent experiments. (**f**) Histological analysis of joint inflammation at day 14 post-LPS injection. Paraffin sections of the limb were stained by HE (upper panel) and Safranin O/Fast green for cartilage (red) staining (lower panel). Scale bar, 60 μm.

ACC4 monoclonal antibody is a mouse ACPA (IgG1) that binds citrullinated Col II, and infusion of this antibody frequently induces collagen antibody-induced arthritis (CAIA) in the presence of the anti-Col II monoclonal antibody M2139 (IgG2b)^11^. To produce sialylated forms of these antibodies, both *mSt6gal1* and *mB4galt1* cDNAs were transfected into ACC4 and M2139 hybridomas (Fig. 4a). The majority of terminal sugars of IgG Fc from double-transfected ACC4 and M2139 hybridoma cells were found to extend to α2,6 sialic acid (G2FS2), whereas those from non-transfected ACC4 and M2139 hybridoma cells were ended with terminal galactose or GlcNAc (G0F or G1F) (Fig. 4b; Supplementary Fig. 15). SNA lectin blot analysis also supported increased Fc-sialylation in the transfectants, whereas SNA binding of the Fab fragment remained unaffected (Supplementary Fig. 16). SNA lectin recognizes Neu5Ac(α2–6)Gal/GalNAc residue found in N-linked and O-linked glycans^36^,^37^; however, the removal of N-linked glycans by PNGase F treatment selectively abolished SNA binding to the Fc fragment, but not to the Fab fragment even under SDS denaturation (Supplementary Fig. 17). The lack of sequence motif for N-linked glycosylation site (N-X-S/T, X is any residues except proline) in the ACC4 Fab domain further supported that SNA binding to the Fab fragment is mediated by either O-linked glycans or other unidentified molecules, rather than by N-linked glycans. Thus, the overexpression of *mSt6gal1* and *mB4galt1* produced IgG antibodies with increased sialylation in an Fc-specific manner. Sialylation of both monoclonal antibodies showed no significant impact on the binding ability to antigens (Fig. 4c). To elucidate the arthritogenic activity of sialylated antibodies, we administered ACC4/M2139 IgG-Sia (+) or ACC4/M2139 IgG into DBA/1 mice to induce CAIA (Fig. 4d). As previously reported, the infusion of ACC4/M2139 IgG induced CAIA in approximately half of DBA/1 mice^11^. However, ACC4/M2139 IgG-Sia (+) failed to induce CAIA in all mice (Fig. 4e). Likewise, histopathological analysis of the joints revealed no signs of inflammation in mice infused with ACC4/M2139 IgG-Sia (+), whereas those with ACC4/M2139 IgG showed the expected arthritis phenotype, i.e., infiltration of monocytes, lymphocytes and granulocytes (Fig. 4f). These results clearly demonstrate that sialylation reduces the pathogenicity of anti-Col II antibodies, including ACPAs.

### Sialylated anti-Col II antibody exhibits regulatory activity

**Figure 5:**
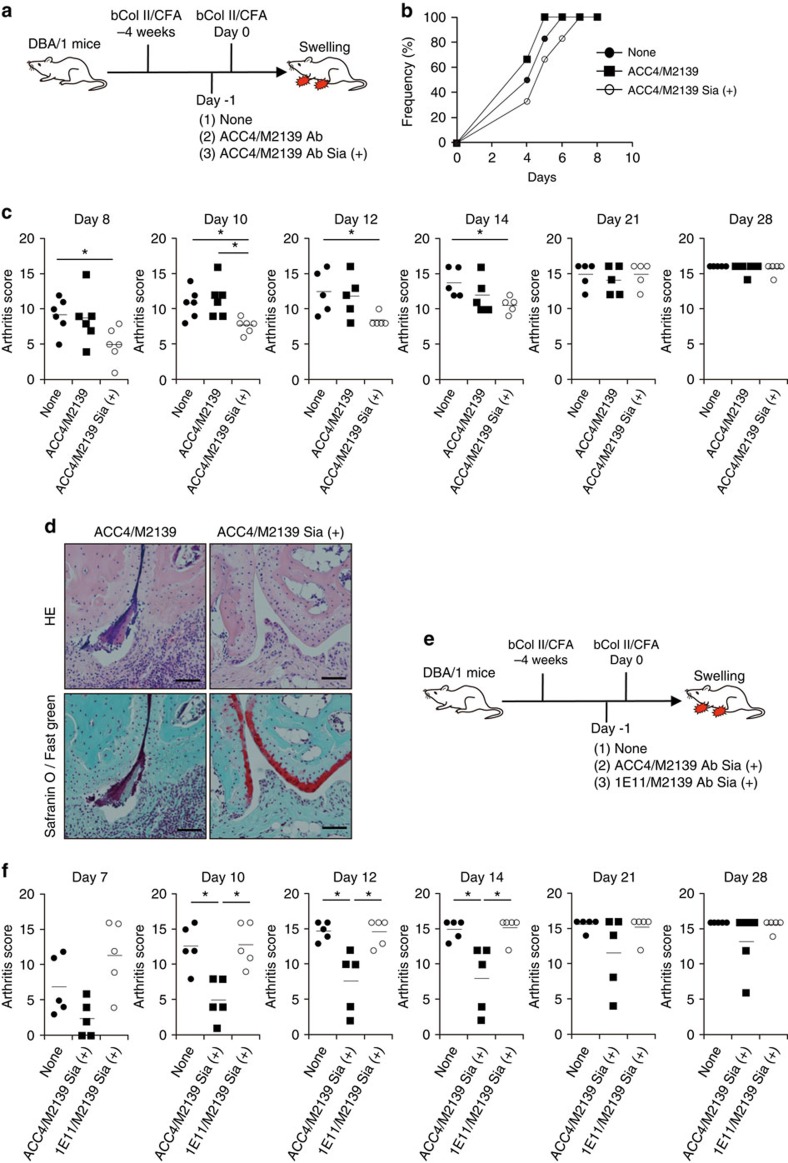
Sialylated ACPAs exhibit antigen-specific regulatory activity in CIA model. (**a**) An experimental scheme to evaluate the regulatory activity of infused antibody is presented. (**b**,**c**) Frequency (**b**) and score (**c**) of arthritis are plotted. Each symbol represents the result from an individual mouse. Data are representative of three independent experiments; these were analysed by Steel–Dwass non-parametric test (*P<0.05). (**d**) Histological analysis of joint inflammation at day 14 after booster immunization. Paraffin sections of the limb were stained by HE (upper panel) and Safranin O/Fast green for cartilage (red) staining (lower panel). Scale bar, 60 μm. (**e**) An experimental scheme to evaluate the regulatory activity of infused antibody. (**f**) Arthritis score of each group of mice is presented. Each symbol represents the result from an individual mouse. Mean values were presented as bars. The data are representative of three independent experiments. Data were analysed by Steel–Dwass non-parametric test (*P<0.05).

Sialylation has been shown to enhance the regulatory activity of IVIG in a K/BxN serum-mediated arthritis model^22^. This prompted us to assess the regulatory activity of sialylated ACPAs in the RA model. We utilized the CIA model because this model mimics both early autoimmune responses and later antibody-dependent effector phase that triggers the development of joint inflammation. In these experiments, some groups of mice were infused with a cocktail of ACC4/M2139 antibody with or without sialylation one day before boosting for CIA induction (Fig. 5a). Although the infusion of sialylated ACC4/M2139 antibodies reduced the incidence of CIA and that of control antibodies increased the incidence, the difference did not reach statistical significance (Fig. 5b). However, mice treated with sialylated antibodies showed significantly milder arthritis scores than untreated mice or those treated with control antibodies (Fig. 5c,d). To estimate whether disease severity correlates with sialylation levels of anti-Col II IgG antibodies in serum, we compared the sialylation levels of anti-Col II IgG from mice infused with ACC4/M2139 (Fig. 5) and ST6Gal1-deficient mice (Fig. 3) on day 7 after CIA induction (Supplementary Fig. 18). Infusion of control ACC4/M2139 did not alter the sialylation levels of anti-Col II IgG in serum; however, infusion of sialylated forms of ACC4/M2139 significantly increased the sialylation levels of anti-Col II IgG. Additionally, the sialylation levels of anti-Col II IgG from ST6Gal1-deficient mice were below the detection limit. The levels were even lower than those in the control mice without antibody infusion, consistent with the data depicted in Fig. 3b. Thus, sialylation not only reduces the arthritogenic activity of anti-Col II antibodies, including ACPAs, but also induces the regulatory activity in the CIA model.

Sialylated IVIG inhibits K/BxN serum-induced arthritis and other autoimmune diseases more potently than non-sialylated IVIG^22^,^34^,^38^,^39^. Thus, the observed regulatory effects of sialylated ACPAs might simply represent antigen-non-specific processes as provided by IVIG. To address this issue, we sialylated the isotype-matched mouse IgG1 antibody, namely, 1E11 (influenza hemagglutinin specific). IgG1 Fc of both ACC4 and 1E11 were heavily sialylated to similar levels after the introduction of *mSt6gal1* and *mB4galt1* genes into the hybridomas (Supplementary Fig. 19). Sialylated ACPA or irrelevant IgG (1E11) was co-infused with M2139 and the regulatory activity of these antibodies was examined (Fig. 5e). M2139 was used in both infusions so that the specific effect of the ACPA could be evaluated. Strikingly, a cocktail of sialylated 1E11/M2139 did not show any regulatory activity at all, while that of sialylated ACC4/M2139 did, as measured by the severity of the arthritis score (Fig. 5f). Thus, these data demonstrate that the regulatory activity shown by sialylated ACPA is based on an antigen-specific event.

## Discussion

Many pieces of clinical evidence suggest a pathogenic role for ACPAs in RA. Before the onset of arthritis, ACPAs display several unique features that are potentially involved in disease pathogenesis, including degalactosylation of IgG Fc, reduced affinity maturation and epitope spreading^40^,^41^,^42^. Unfortunately, there is no RA mouse model available to completely mimic ACPA-mediated RA pathogenesis. Therefore, we evaluated the pro- and anti-inflammatory functions of IgG Fc glycans in CIA mouse model, and found that sialylation level is crucial in the control of RA pathogenesis. Furthermore, highly sialylated collagen antibodies including ACPA was successfully applied as an immunosuppressive drug ameliorating joint inflammation in an CIA mouse model. Thus, the modulation of Fc glycosylation patterns of RA-associated IgG may be a key target for the development of antigen-specific immunotherapies for RA.

Pro- and anti-inflammatory functions of IgG Fc with different sialylation patterns have been addressed in antibody infusion systems which mirror the effector function of IgG-mediated inflammatory diseases, including RA^19^,^22^,^34^,^43^,^44^,^45^,^46^,^47^. However, because of the lack of other autoimmune components, this system does not replicate all features of RA pathogenesis. Here, we tested a genetic approach, in which the sialylation of endogenous IgG was blocked by conditional ST6Gal1 deficiency. The function of IgG sialylation was assessed in the CIA mouse model where pathogenic antibodies are produced along with other important autoimmune components. We noted that desialylation exacerbated RA joint inflammation without affecting antigen-specific IgG production; therefore, severe bystander effects on antigen-specific B-cell development were unlikely. Thus, our data strongly support a proinflammatory function of desialylated IgG on the development of RA.

Although RA patients showed reduced galactosylation and a resulting reduction of sialylated forms, two mouse RA models showed a specific reduction in sialylation, but not in galactosylation. In the absence of animal models with an appropriate genetic background and autologous Col II, it is difficult to fully reproduce the long-lasting chronic inflammation observed in RA patients^48^,^49^. Therefore, the differential IgG glycosylation patterns between RA patients and mouse models may be the result of long-term chronic exposure to inflammation in RA patients, leading to broader effects on the glycosylation state of immune cells. A key enzyme for IgG galactosylation is B4-galactosyltransferase (GTase)^50^ which exists in both intracellular and extracellular compartments^51^,^52^,^53^. Intracellular GTase activity was significantly reduced in lymphocytes from RA patients, in a manner proportional to disease severity^54^, suggesting that long-lasting chronic inflammation may cause a reduction in GTase levels in B cells and promote the degalactosylation of IgG. In any case, the consequences of both degalactosylation and desialylation manifest as reduced sialylation, which we show is sufficient to exacerbate RA pathogenesis. However, it is still possible that degalactosylation itself can increase the proinflammatory activity of human ACPAs through pathways other than desialylation in RA patients.

Importantly, sialylated ACPAs acquired regulatory activity in CIA in an antigen-specific manner, while the sialylation of irrelevant IgG was non-preventive. It should be stressed that ACPAs, but not irrelevant IgG, are able to react with citrullinated proteins in the joints^10^; therefore, it is expected that sialylated ACPAs would be more concentrated in diseased tissue. In line with this, we propose three possible models: (1) competition for antigen-binding sites with desialylated ACPA that trigger inflammatory reactions by binding to ‘activating' FcγRs or analogous receptor molecules in the joints (space competition model), (2) direct activation of inhibitory FcγRIIb on joint macrophages (direct suppression model) and (3) indirect induction or activation of inhibitory FcγRIIb via other ligand molecules or ligand-expressing cells present in the joints, as observed for anti-inflammatory activity of IVIG (indirect suppression model)^44^. The first model is supported by previous findings that sialylation of IgG reduced the binding affinity to all activating FcγRs^43^,^44^; thus, the replacement of local ACPAs by sialylated types would reduce the average pathogenic activity of local ACPAs. The third model is analogous to the model proposed by Ravetch's group in which the systemic anti-inflammatory effects of sialylated IVIG are mediated by binding to SIGN-R1 (DC-SIGN in humans) on marginal zone macrophages, and subsequent up-regulation of FcγRIIb on inflammatory macrophages^44^.

An important question remains: how can the sialylation levels of ACPAs be modulated clinically. As shown in this study, one key target molecule is ST6Gal1. This enzyme is ubiquitously expressed in many cell types, including plasma cells^20^; however, it is important to note that ST6Gal1 expression in plasma cells is influenced by the types of antigens and immunization protocols^20^,^21^. In particular, antigens or immunization regimes that do not activate T cells maintain ST6Gal1 expression in plasma cells, leading to the production of highly sialylated IgG, whereas B-cell activation in the presence of T cell turns off the expression of this enzyme^21^. If this is the case during the developmental of IgG ACPAs, then T-cell-independent activation of ACPA^+^ B cells may be a better approach to suppress RA-specific inflammation rather than the current B-cell depletion protocols.

## Methods

### Patients

Blood samples from clinically defined ACPA^+^ or ACPA^−^ RA patients and from healthy donors were collected in Osaka University Hospital, Nagoya University Hospital and University of Tokyo Hospital. Written informed consent was obtained from all participants. This study was approved by the ethical committees of Nagoya University, Osaka University, University of Tokyo, National Institute of Infectious Diseases, National Institute of Health Sciences, and RIKEN Center for Integrative Medical Sciences. Sera were isolated from the blood samples by using Ficoll-Paque PLUS (GE Healthcare Ltd., Chalfont St Giles, UK) with fibrinogen removed using rapid clotting tubes (NIPRO, Osaka, Japan), and stored at −80 °C.

### Mice

Male DBA/1 and C57BL/6 mice (8-week old) were obtained from SLC Inc. (Hamamatsu, Japan). Transgenic mice expressing Cre recombinase under the control of activation-induced cytidine deaminase gene promoter (hereafter, AID-Cre mice) were provided by R. Casellas (National Institute of Arthritis and Musculoskeletal and Skin Diseases). HLA-DR4 transgenic mice were obtained from Taconic Biosciences. All experimental protocols were approved by the animal experimental committees of the Graduate School of Medicine in Nagoya University and Osaka University.

### Generation of ST6Gal1^f/f^ AID-Cre mice

To generate ST6Gal1^f/f^ AID-Cre mice, three fragments were subcloned from *mSt6gal1* locus in C57BL/6 strain-derived RPCI23.C BAC clone (ID, 215I11) and these were inserted into a targeting vector containing three *loxP* sites, two *FRT* sites and neomycin (*Neo*) and diphtheria toxin (*DT*) cassettes (Supplementary Fig. 11). An exon 4 fragment of *mSt6gal1* encoding the catalytic domain L motif was inserted between two *loxP* sites in the targeting vector. Eighty micrograms of linearized targeting vector was electroporated into a C57BL/6 embryonic stem (ES) cell line (B6J-S1UTR line), and G418-resistant transfectants (300 μg ml^−1^) were selected. ST6Gal1-floxed allele-containing clones were screened by three patterns of PCR for homologous recombination. Chimera mice were generated by aggregation of ST6Gal1-floxed allele-containing ES cells with ICR mouse embryos and by transfer into surrogate mice. Chimera mice were mated with C57BL/6 mice, and the genotypes of the offspring were screened for the ST6Gal1-floxed allele. Heterozygous ST6Gal1^f/+^ mice were mated with Flp mice to delete the neo cassette. Finally, they were crossed with AID-Cre mice to establish ST6Gal1^f/f^ AID-Cre mice. For CIA study, both male and female mice (12–17-week old) were used.

### Separation and purification of serum antibodies

CCP1 (HQCHQESTXGRSRGRCGRSGS, circled between two cysteines, X: citrulline) and Arg-control peptide (HQCHQESTRGRSRGRCGRSGS, circled between two cysteines) were purchased from Biologia Co. (Nagoya, Japan). Chicken or bovine type II collagen (Col II) and human fibrinogen (Fib) were purchased from Sigma-Aldrich (St Louis, MO, USA). The above-described antigens were conjugated to CNBr-activated Sepharose 4B (GE Healthcare Ltd.) according to the manufacturer's protocol. After ammonium sulfate precipitation, human and mouse sera were loaded on the columns. Column-binding antibodies were eluted by 0.17 M glycine-HCI (pH 2.7). Non-binding fractions were also collected as flow-through and then further loaded on HiTrap Protein G columns (GE Healthcare Ltd.) for IgG purification. The purity of IgG fractions was confirmed by ELISA. To collect CCP2- and Col II-binding antibodies for western blotting analysis, 50 μl (for CCP2) and 1 μl (for Col II) of mouse serum were incubated in CCP2-coated plates (MBL, Nagoya, Japan) or Col II-coated (10 μg ml^−1^) plates overnight at 4 °C. After washing with phosphate-buffered saline (PBS), bound antibodies were eluted with 0.1 M glycine pH 2.7 and neutralized with 1 M Tris-HCI pH 9.0. The amount of eluted antibodies was evaluated by western immunoblotting analysis. Original images are presented in Supplementary Fig. 20.

### ELISA

To quantitate the amounts of antibodies, plates were coated with 10 μg ml^−1^ Fib (Sigma-Aldrich), 10 μg ml^−1^ Col II (Sigma-Aldrich), 20 μg ml^−1^ CCP1, 20 μg ml^−1^ Arg-control peptide, 5 μg ml^−1^ anti-mouse IgG F(ab)^2^ fragments (Sigma-Aldrich), or 5 μg ml^−1^ anti-human IgG F(ab)^2^ fragments (Sigma-Aldrich) in 0.1 M carbonate buffer (pH 9.0) overnight at 4 °C. After blocking with 1% BSA in PBST (PBS containing 0.1% Tween-20), serially diluted sera or purified IgGs were added to each well and the amount of bound antibodies was detected using HRP-conjugated secondary antibodies. Antibodies used for secondary antibodies were as follows: goat anti-mouse IgG-HRP (SouthernBiotech., Birmingham, AL, 1030-05), goat anti-mouse IgG2c-HRP (SouthernBiotech, 1090-05) and goat anti-human IgG-HRP (SouthernBiotech, 2040-05).

### Collagen-induced arthritis

Complete Freund's adjuvant (CFA) was prepared by resuspending 5 mg of desiccated *Mycobacterium tuberculosis* H37Ra debris (BD Biosciences, San Jose, CA, USA) in 1 ml of incomplete Freund's adjuvant (BD Biosciences). Col II (2 mg ml^−1^), from either bovine or chicken, was emulsified with CFA (Col II:CFA=1:1) and then intradermally injected twice at the base of the tail at intervals of 4 weeks. Mice were monitored daily for swelling of limbs for at least 28 days and serum samples were collected at day 7 or 14 after boosting.

### Enforced sialylation of mouse monoclonal IgG

ACC4 and M2139 hybridomas secreting mouse ACPA (IgG1) and anti-Col II (IgG2b) mAb, respectively, were provided by R. Holmdahl (Lund University). 1E11 hybridoma secretes mouse IgG1 mAb against influenza hemagglutinin. This hybridoma was established in our laboratory from BALB/c mice which were hyperimmunized with H3N2 influenza virus (X31 strain). Both *mSt6gal1* and *mB4galt1* cDNAs were transfected into ACC4, M2139 and 1E11 hybridomas by using the retrovirus system, in which pMXs-IRES-EGPR/pMSCV-IRES2-DsRed express2 vectors and GP2-293 packaging cells were used. GFP- and DsRed-positive hybridoma cells were sorted at least twice by using FACS Aria II (BD Biosciences), and the culture supernatant of purified hybridomas was subjected to IgG purification. The quality and quantity of purified IgGs were confirmed by Coomassie brilliant blue staining of SDS–PAGE (polyacrylamide gel electrophoresis) gels and ELISA.

### Lectin blotting analysis of IgG Fc

Purified IgG (5 μg) was incubated in digestion buffer (10 mM EDTA, 10 mM cysteine in PBS (pH 7.4)) with 0.25 μg of papain (Sigma-Aldrich) for 2 h at 37 °C. Digested IgGs were separated by 10% SDS–PAGE (IgGs, 1.25 μg per lane for lectin blotting; 100 ng per lane for western blotting), and transferred onto polyvinylidene difluoride membranes by semi-dry electrophoresis. For western or lectin blotting, the membranes were incubated with either goat anti-mouse IgG-HRP (Cell Signaling Technology Japan, K.K., Tokyo, Japan, #7076) or biotin-conjugated lectin (Vector Laboratories, Inc., Burlingame, CA). Terminal α2,6 sialic acid (Sia), β1,4 galactose (Gal), *N*-acetylglucosamine (GlcNAc), and α-linked mannose (αMan) were detected by SNA (B-1305), ECL (B-1145), GSL II (B-1215) and ConA (B-1005S) lectin, respectively.

### PNGase F treatment

Purified IgGs (10 μg) from ACC4 hybridomas were digested by papain. Digested IgGs were denatured by heating at 100 °C for 10 min in presence of 1% SDS. Denatured IgGs were neutralized by Nonidet P-40 and incubated with or without 1 unit per μl PNGase F (Roche) for 24 h at 37 °C. Then, PNGase F-treated IgG was subjected to western or lectin blotting as described above.

### Collagen antibody-induced arthritis

DBA/1 mice (10–12-week old) were administered by intraperitoneal injections of both ACC4 and M2139 (4.5 mg each) with or without sialylation. To induce arthritis, the mice were intraperitoneally injected with 25 μg of LPS from *Escherichia coli* 055:B5 (Sigma-Aldrich) 5 days later. Mice were monitored daily for swelling encompassing the ankle, foot and digits or ankylosis of the limb for at least 28 days and serum samples were collected at day 7 after boosting.

### Histological analysis of knee joints

Naive and arthritis-induced mice were sacrificed and perfused with PBS, followed by 4% paraformaldehyde (PFA) in PBS. All limbs were removed and post-fixed with 4% PFA in PBS for 1 week, decalcified in 10% EDTA in Milli-Q water (pH 7.4) for 30 days at 4 °C, dehydrated in a graded ethanol series, and embedded in paraffin. Blocks were cut into serial sagittal sections (4.5 μm). Each section was stained with hematoxylin/eosin (HE) or Safranin O/Fast Green for cartilage staining and observed by light microscopy.

### LC-ESI-MS analysis

Aliquots of IgG fractions (5–10 μg) were carboxymethylated and digested by trypsin. Digested samples from RA patients and mouse model were analysed by an LC-ESI-MS system consisting of a Paradigm MS4 (Michrome Bioresources, Inc.) or an EASY-nLC 1000 (Thermo Scientific) HPLC system and Orbitrap Elite hybrid (Thermo Scientific, Inc.) or Q Exactive (Thermo Scientific, Inc.) MS system equipped with a nanoESI ion source, and digested samples from ACC4, M2139 and 1E11 hybridoma IgGs were analysed by an LC-ESI-MS system consisting of an EASY-nLC 1000 (Thermo Scientific) and Orbitrap Fusion Tribrid mass spectrometer (Thermo Scientific, Inc.). Details of the method used for LC-ESI-MS analysis are described in Supplementary Methods.

### MALDI-TOF-MS analysis

Samples for MALDI-TOF-MS were prepared from 10 μg of purified IgGs from CIA-induced ST6Gal1^f/f^ AID-Cre, ST6Gal1^f/f^, AID-Cre and C57BL/6j mice and total serum IgG from ST6Gal1 KO mice by BlotGlyco (Sumitomo Bakelite Co., Tokyo, Japan) according to the manufacturer's protocol^55^. Samples were analysed by MALDI-TOF system by using an Autoflex III TOF/TOF mass spectrometer equipped with a reflector and controlled by the FlexControl 3.0 software package (Bruker Daltonics GmbH, Bremen, Germany). Details of the method used for MALDI-TOF-MS analysis are described in Supplementary Methods.

### Real-time RT–PCR

The indicated tissues were isolated from mice and homogenized in Trizol (Life Technologies). Total RNA was extracted from tissues with Trizol according to the manufacturer's protocol and then reverse-transcribed into cDNA by using M-MLV Reverse Transcriptase (Life Technologies) and oligo dT primer (Sigma-Aldrich). Real-time RT-PCR (PCR with reverse transcription) was performed using 4 ng of RNA/cDNA per well, with F-400 from an SYBR green qPCR kit (Finnzymes, Espoo, Finland), and Thermal Cycler PTC-20 (Bio-Rad Laboratories, Inc., Hercules, CA, USA). The PCR conditions were as follows: preheating for 10 min at 95 °C, 40 cycles of 95 °C (10 s), 60 °C (20 s) and 72 °C (20 s). The plate reader was set at 75 °C (2 s) depending on individual primer pairs. *mSt6gal1* or *mSt6gal2* cDNA vectors were used as the standard. Every sample was measured in duplicate, and gene expression levels were analysed by using Opticon Moniter3 software (Bio-Rad Laboratories).

### Statistical analysis

Data were presented as individual donor, mouse or samples, and mean values were presented as bars. Column graph data were shown as mean±s.d. No statistical method was used to determine sample size. There was no randomization of mice or samples before analysis, and the mice were selected based on availability. No blinding was performed in this study. Donors, mice or samples were not excluded from any of analysis in this study. Results were initially analysed for homogeneity of variance using Bartlett's, Hartley's and Levene's tests. The Shapiro–Wilk test was used to verify that the data followed normal distribution. In the case of data that did not meet the assumptions of homogeneity of variance or normal distribution, non-parametric analysis was used for statistical tests. Statistical significance was calculated by using two-tailed Student's *t* test (parametric analysis) or Mann–Whitney test (non-parametric analysis) for two comparison groups and one-way analysis of variance (ANOVA) followed by Tukey–Kramer *post hoc* test (parametric analysis) or Steel–Dwass test (non-parametric analysis) for >3 comparison groups. The frequency of arthritis between groups was analysed using two-tailed Fisher's exact test on each day. All statistical significances were set at *P<0.05, **P<0.01, ***P<0.001.

## Additional information

**How to cite this article:** Ohmi, Y. *et al.* Sialylation converts arthritogenic IgG into inhibitors of collagen-induced arthritis. *Nat. Commun.* 7:11205 doi: 10.1038/ncomms11205 (2016).

## Supplementary Material

Supplementary Information Supplementary Figures 1-20, Supplementary Tables 1 & 2, Supplementary Methods and Supplementary References.
