# Supplementary material for: Sialylation converts arthritogenic IgG into inhibitors of collagen-induced arthritis
Source: Nat Commun. 2016 Apr 5;7:11205. doi: 10.1038/ncomms11205 (PMC4822049; doi:10.1038/ncomms11205)
Supplement: Supplementary Information — Supplementary Figures 1-20, Supplementary Tables 1 & 2, Supplementary Methods and Supplementary References. [file ncomms11205-s1.pdf]

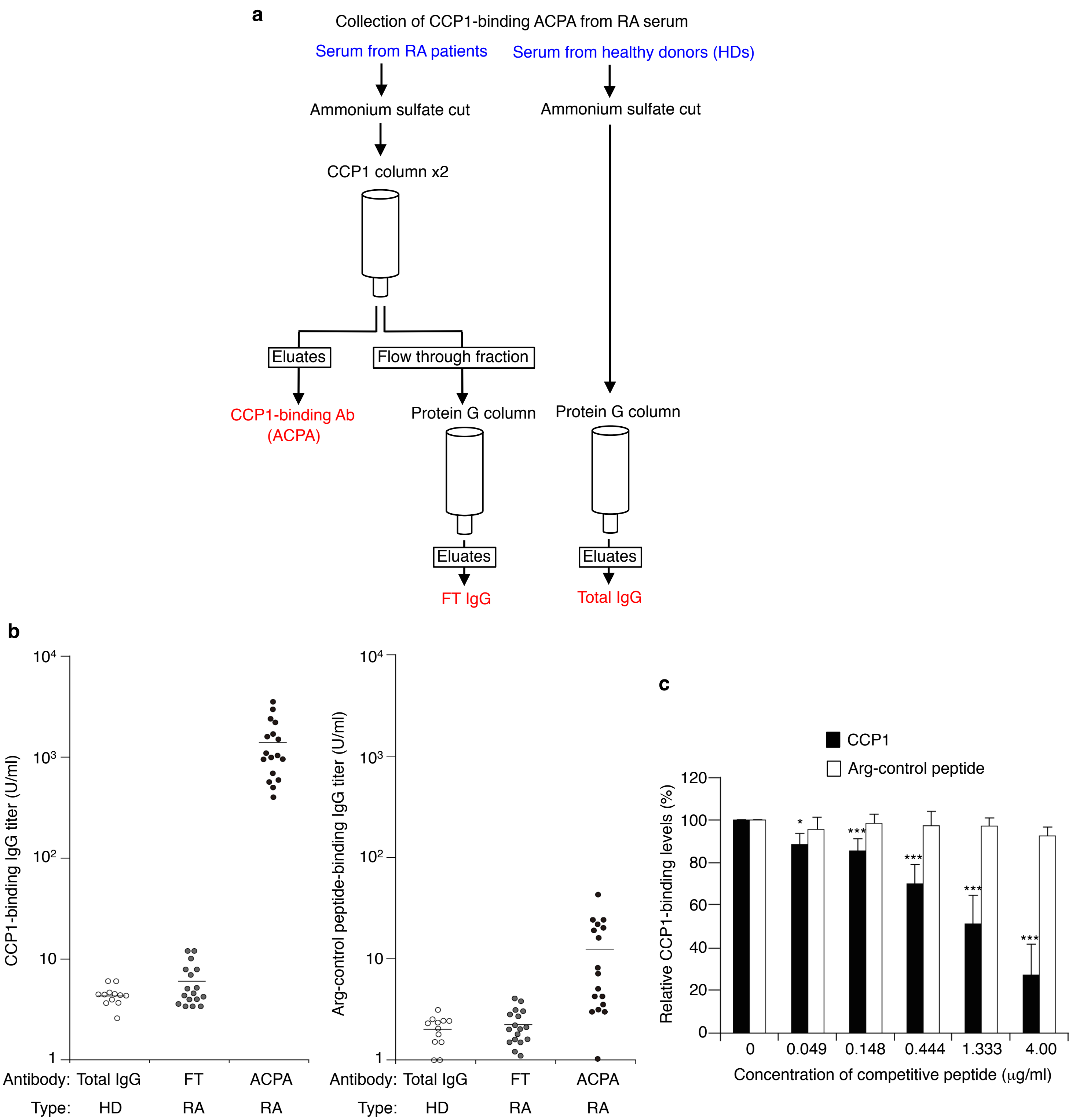


**Supplementary Figure 1. Purification of ACPA and flow through (FT) IgG from RA patients and total IgG from healthy donors (HDs).** (**a**) An experimental scheme for purification of three types of IgG fractions. (**b**) Antibody titers of fractionated IgG against CCP1 and Arg-control peptide are presented. Mean values were presented as bars. Each circle represents the result from an individual donor. (**c**) ELISA plates were coated with 20 μg/ml CCP1 peptide, and then the suboptimal dose of ACPAs were added to ELISA plates after pre-incubating with the indicated doses of competitive peptides. Plates were color-developed with anti-IgG-HRP and results are presented as relative binding to those without competitive peptides. Data are shown as mean ± standard deviation [*n*=6 for both peptides]. Data were analyzed by two-tailed Student's *t* test (**P* <0.05; ****P* <0.001).

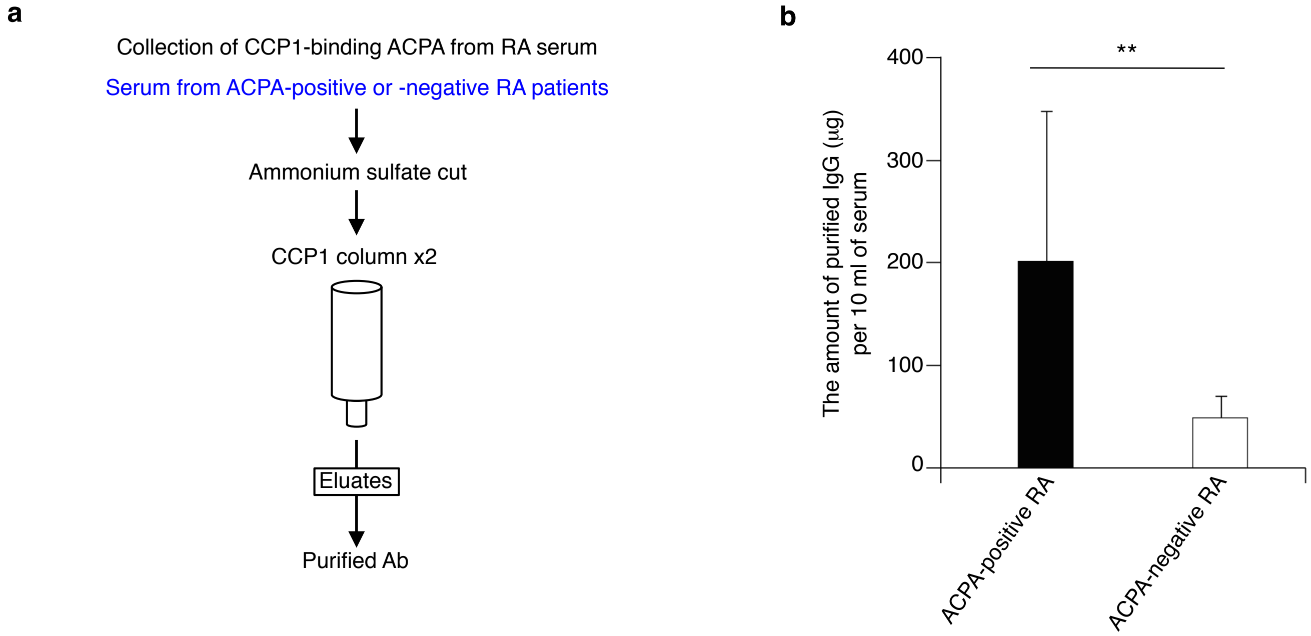


**Supplementary Figure 2. The amounts of IgGs recovered from ACPA-positive and -negative RA patients.** (**a**) An experimental scheme for purification of CCP1-binding antibodies from ACPA-positive and –negative patients. (**b**) The amounts of IgGs recovered from each group are presented. [$n$=17 for ACPA-positive RA, $n$=5 for ACPA-negative RA]. Data are shown as mean ± standard deviation. Data were analyzed by Mann-Whitney non-parametric test (**$P$ <0.01).

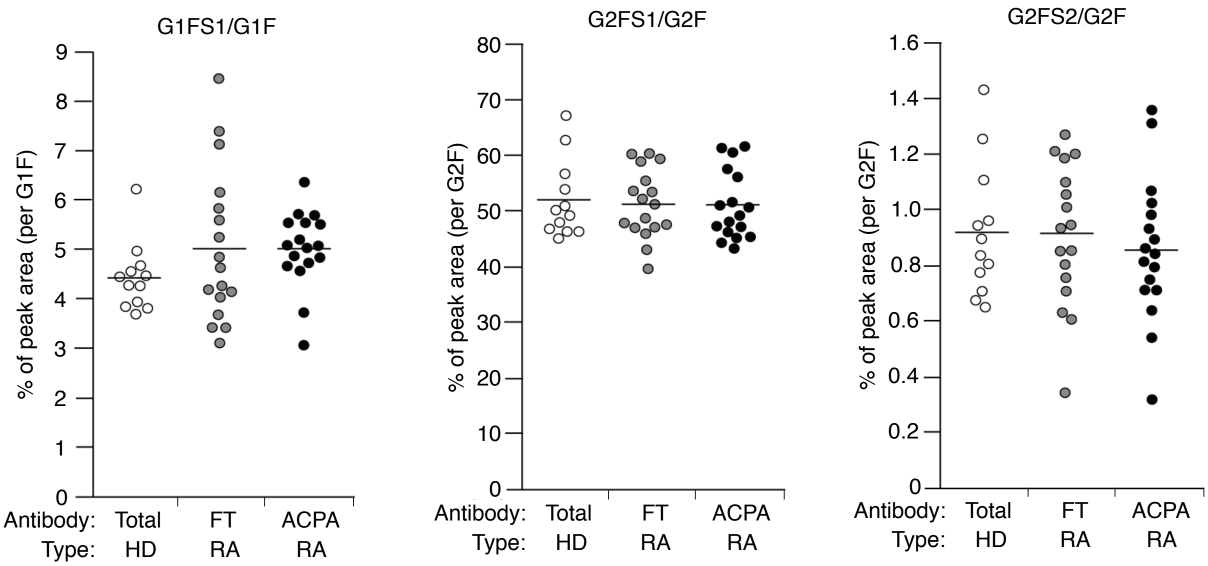


**Supplementary Figure 3. Ratios of sialylated IgG1 Fc to galactosylated IgG1 Fc in the total serum of HDs (Total/HD), FT of CCP columns (FT/RA) and CCP-binding ACPA (ACPA /RA) from RA patients.** Ratios of sialylated IgG1 Fc to galactosylated IgG1 Fc in each group of serum were calculated and plotted as Figure 1. To normalize the variability, relative peak areas of G1F [11] or G2F [15] were deliberately set at 100%. Mean values were presented as bars. Each circle represents the result from an individual donor.

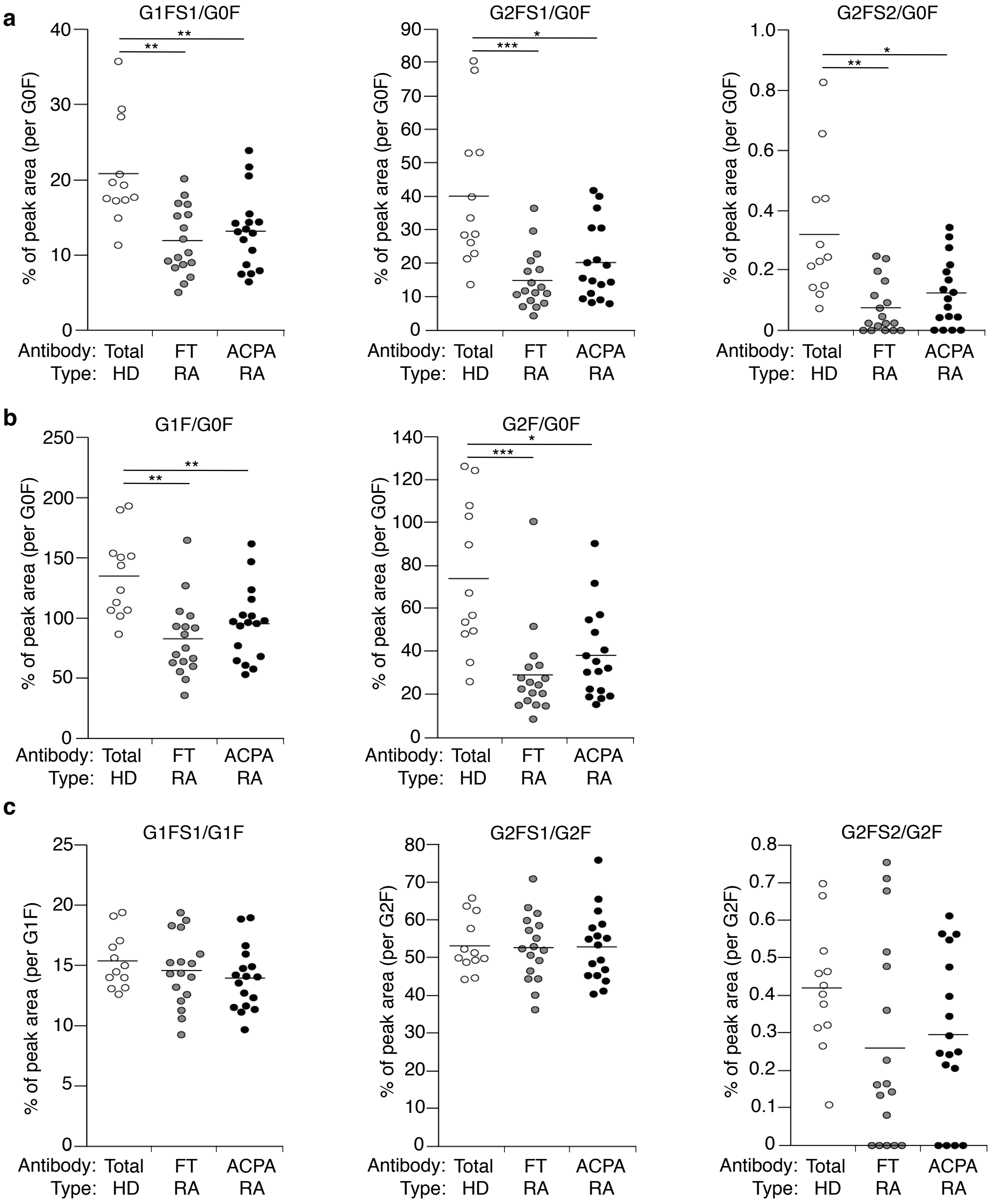


**Supplementary Figure 4. LC-ESI-MS profiles of IgG2 Fc glycans in the total serum of HDs (Total/HD), FT of CCP columns (FT/RA) and CCP-binding ACPA (ACPA/RA) from RA patients.** (**a** and **b**) Ratios of sialylated (**a**) and galactosylated (**b**) IgG Fc to agalactosylated IgG Fc in each group of serum were calculated and plotted. (**c**) Ratios of sialylated IgG Fc to galactosylated IgG Fc in each group of serum were calculated and plotted. Each circle represents the result from an individual donor. Data were analyzed by Steel-Dwass non-parametric test (**P* <0.05; ***P* <0.01; ****P* <0.001).

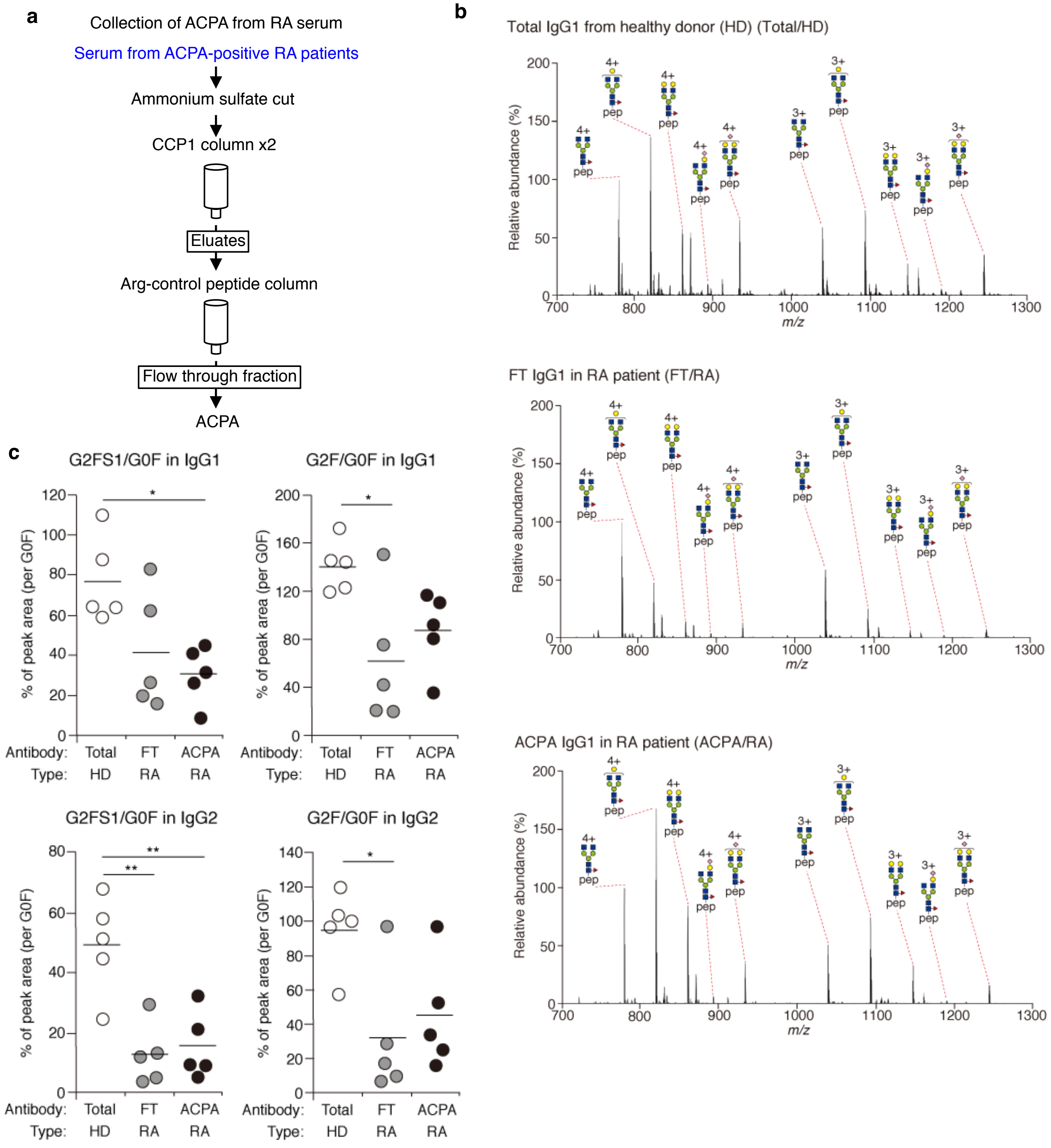


**Supplementary Figure 5. LC-ESI-MS profiles of IgG1 and IgG2 Fc glycans in the ACPA (ACPA/RA) from RA patients.** (**a**) An experimental scheme for purification of ACPA fractions to eliminate sticky molecules. (**b**) Mass spectrometry profiles in each group donors. Relative abundance of each peak was calculated from monoisotopic mass by setting the number of $G0F^{4+}$ of IgG1 as 100%. pep, peptide moiety (TKPREEQYNSTYR). (**c**) Peak area ratios of sialylated and galactosylated IgG1 and 2 Fc glycans to agalactosylated IgG1 and 2 Fc glycans (G0F) in each group of serum were calculated and plotted. Each circle represents the result from an individual donor. Mean values were presented as bars. Data were analyzed by one-way analysis of variance (ANOVA) with Tukey-Kramer *post hoc* test (**P* <0.05; ***P* <0.01).

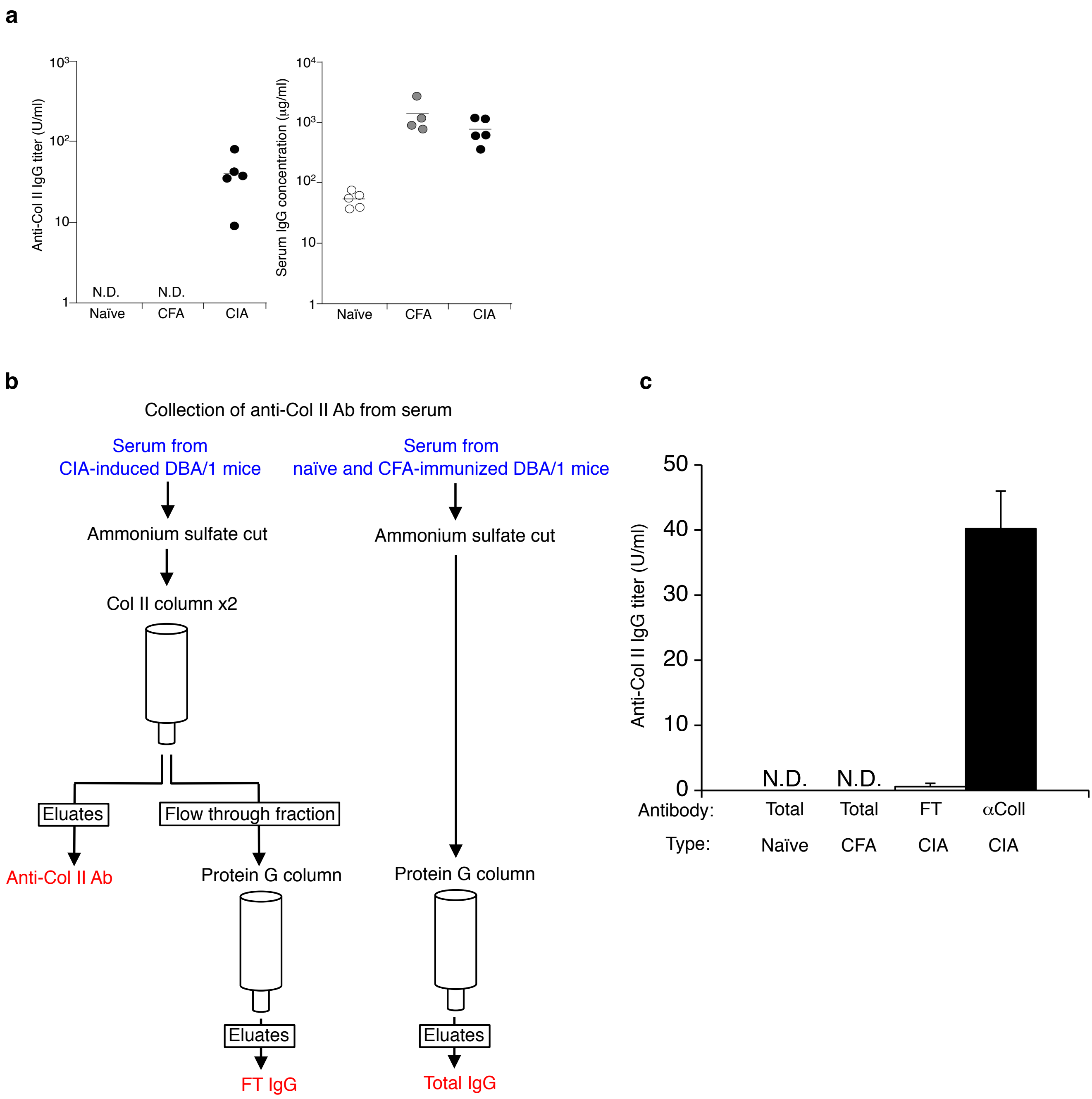


**Supplementary Figure 6. Purification of total serum IgG from naïve or CFA-immunized DBA/1 mouse, and anti-Col II IgG or FT IgG from CIA-induced DBA/1 mouse.** (**a**) Anti-Col II IgG titer and total serum IgG concentration in each group of serum is presented. Each circle represents the result from an individual mouse. N.D., not detectable. (**b**) An experimental scheme for purification of three types of IgG fractions. (**c**) Anti-Col II IgG titers are presented. N.D., not detectable. Data are shown as mean ± standard deviation [*n*=5 for αCol II/CIA, *n*=5 for FT/CIA, *n*=4 for Total/CFA, *n*=5 for Total/naïve].

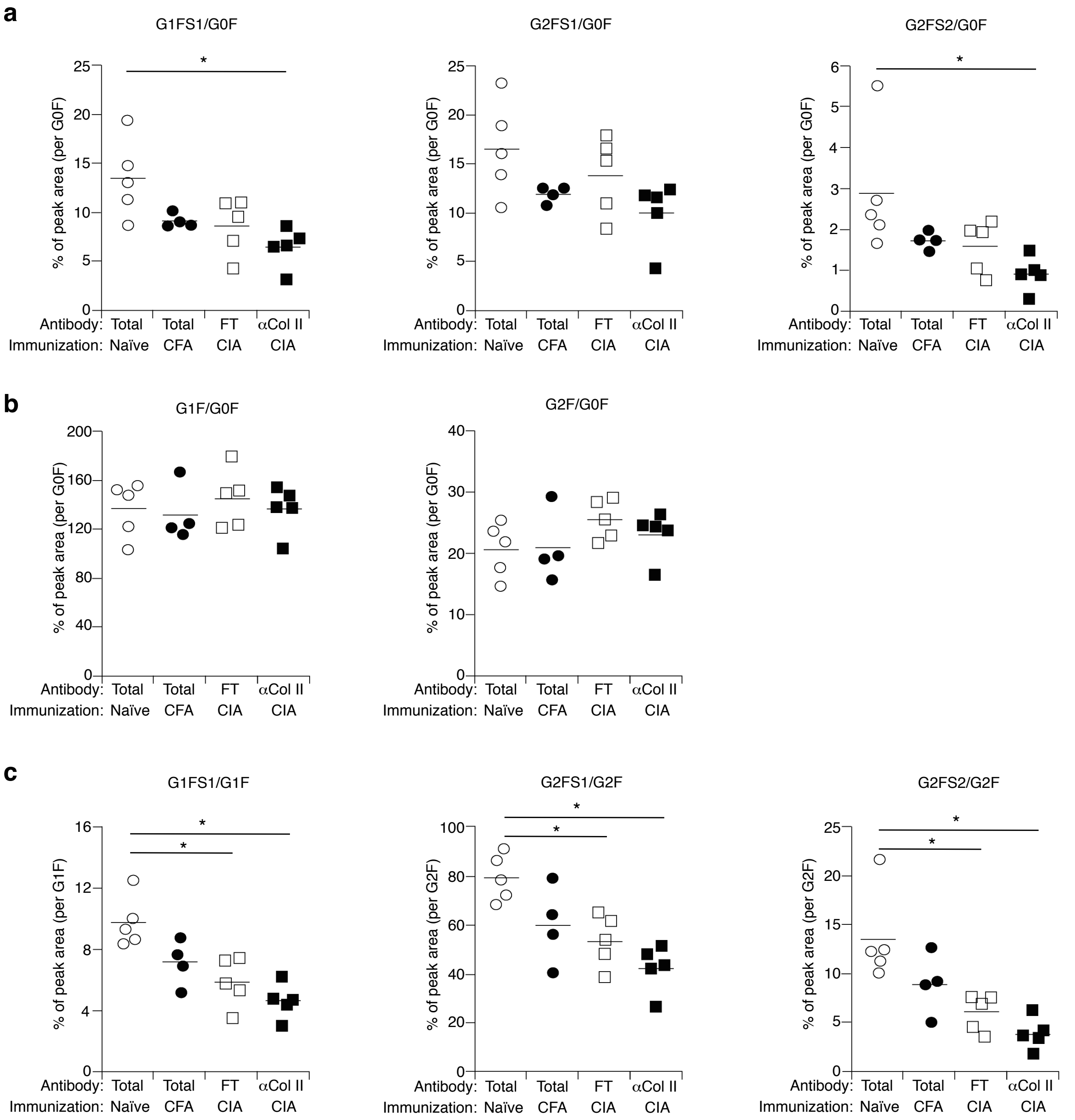


**Supplementary Figure 7. LC-ESI-MS profiles of IgG2a/b Fc in the serum of naïve (Total/naïve) or CFA-immunized DBA/1 mouse (Total/CFA), and FT of Col II column (FT/CIA) or anti-Col II IgG (αCol II/CIA) from CIA-induced DBA/1 mouse.** (**a** and **b**) Ratios of sialylated (**a**) and galactosylated (**b**) IgG Fc to agalactosylated IgG Fc from each group of serum were calculated and plotted. (**c**) Ratios of sialylated IgG Fc to galactosylated IgG Fc in each group of serum were calculated and plotted. Mean values were presented as bars. Each symbol represents the result from an individual mouse. Data were analyzed by Steel-Dwass non-parametric test (*$P$ <0.05).

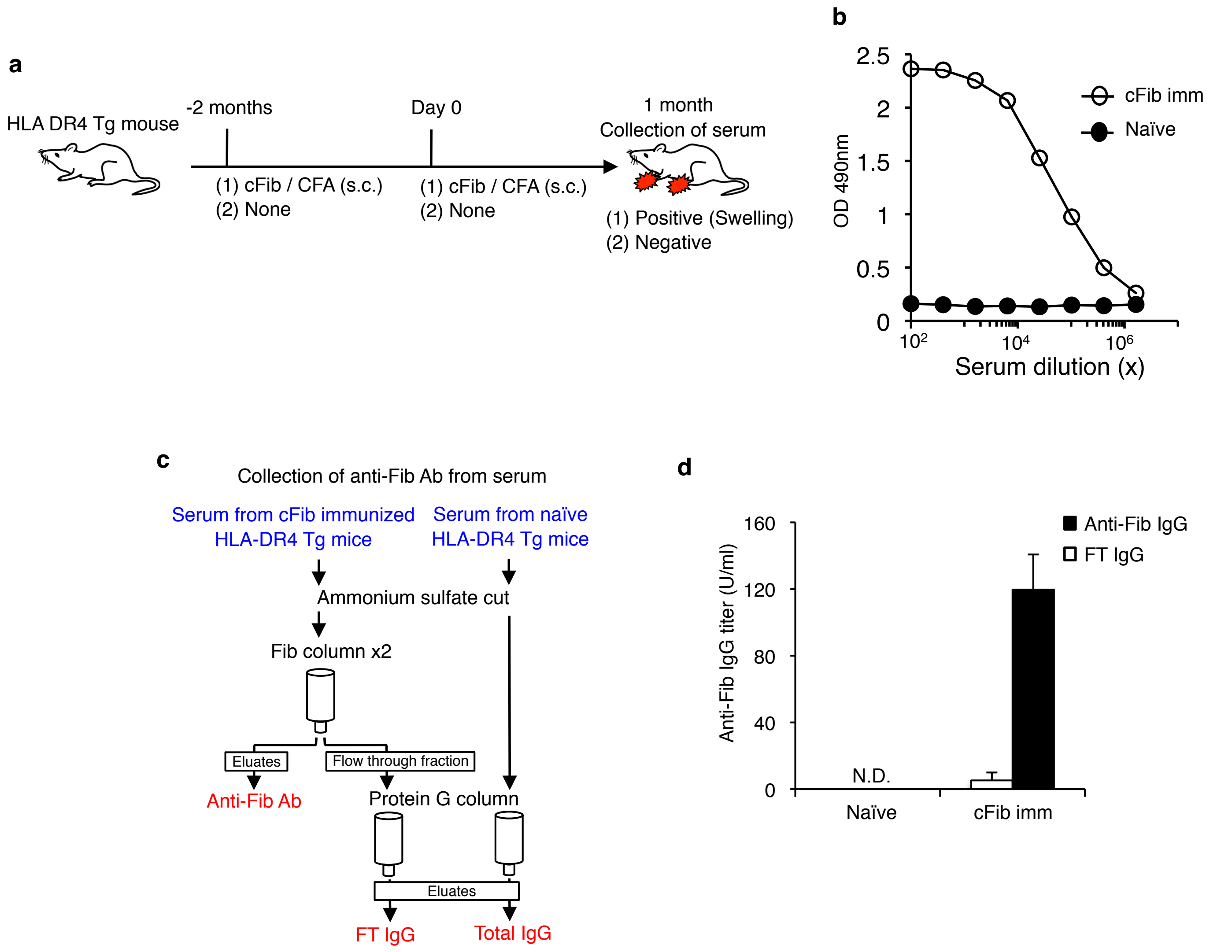


**Supplementary Figure 8. Purification of total serum IgG from naïve HLA-DR4 transgenic (Tg) mouse and anti-fibrinogen (Fib) IgG and FT IgG from citrullinated fibrinogen (cFib)-immunized HLA-DR4 Tg mouse.** (**a**) An experimental scheme for cFib-induced arthritis in HLA-DR4 mice. Arthritis was induced by cFib immunization. (**b**) Anti-Fib IgG titers in naïve and cFib-immunized mice was compared by ELISA. (**c**) An experimental scheme for purification of three types of IgG fractions. (**d**) Anti-Fib IgG titers of purified IgGs are presented. N.D., not detectable. Data are shown as mean ± standard deviation [*n*=5 for all types of IgG].

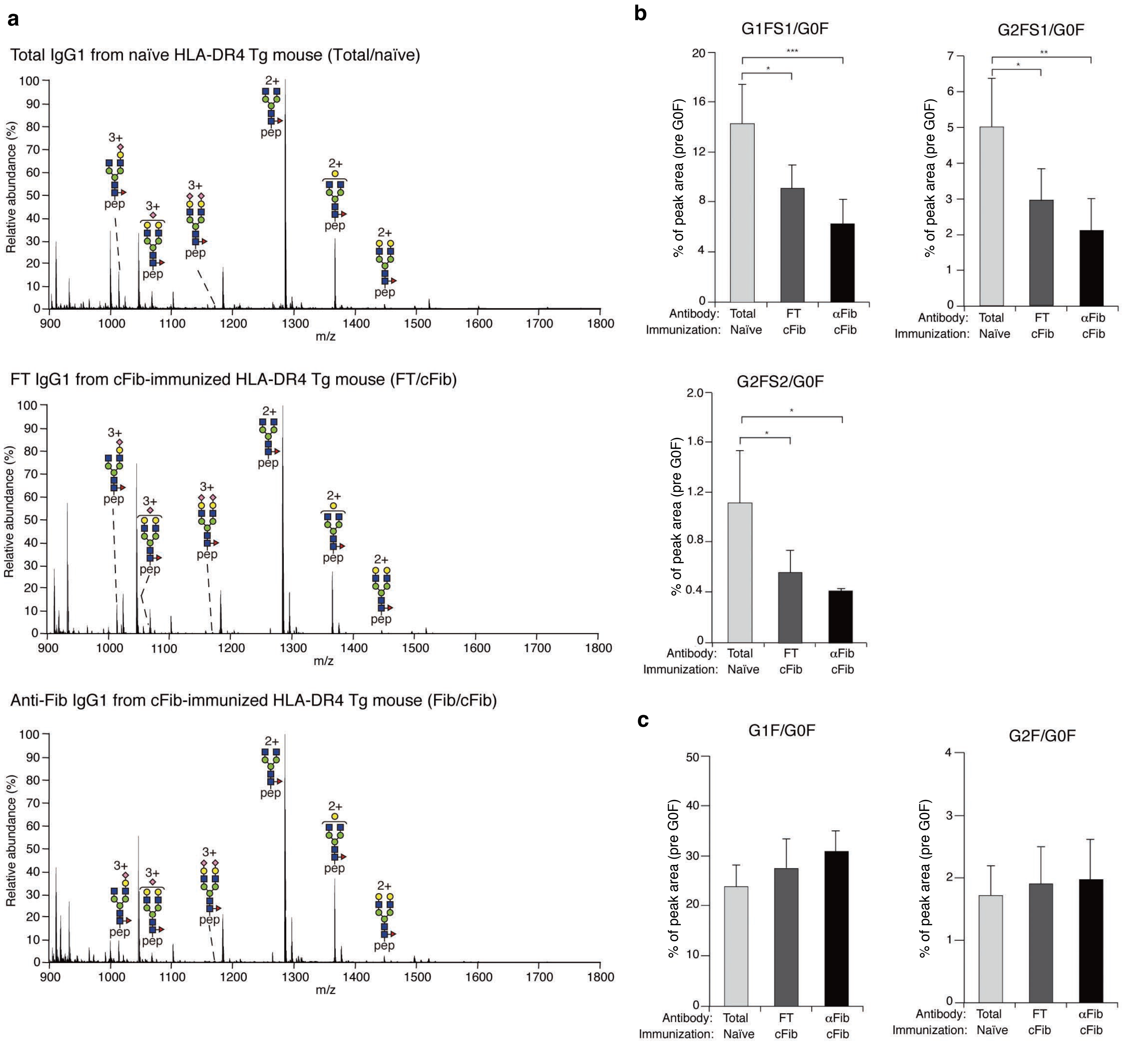


**Supplementary Figure 9. LC-ESI-MS analysis of IgG1 Fc glycans in total serum from naïve HLA-DR4 Tg mouse (Total/naïve), and flow-through of Fib column (FT/cFib) or anti-Fib IgG1 (Fib/cFib) from cFib-immunized HLA-DR4 Tg mouse.** (**a**) Mass spectrometry profiles in each group mouse. Relative abundance of each peak was calculated from monoisotopic mass by setting the number of $G0F^{2+}$ as 100%. (**b** and **c**) Ratios of sialylated (**b**) or galactosylated (**c**) IgG Fc glycans to agalactosylated IgG Fc glycan (G0F) in the indicated groups were calculated. Data are shown as mean ± standard deviation [*n*=5 for all types of IgG1]. Data were analyzed by one-way analysis of variance (ANOVA) with Tukey-Kramer *post hoc* test (*$P$ <0.05; **$P$ <0.01; ***$P$ <0.001).

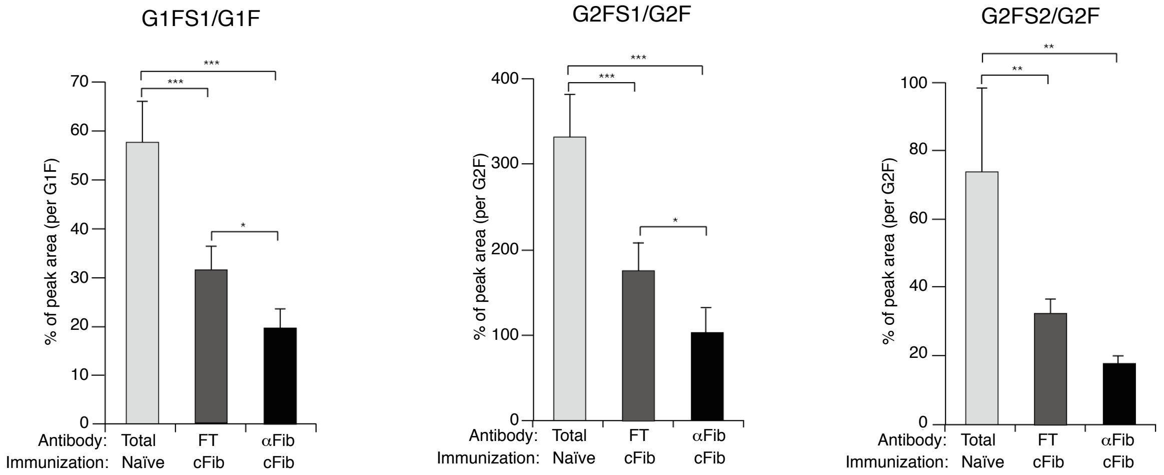


**Supplementary Figure 10. Ratios of sialylated IgG Fc to galactosylated IgG1 Fc in total serum from naïve HLA-DR4 Tg mouse (Total/naïve), and flow-through of Fib column (FT/cFib) or anti-Fib IgG1 (Fib/cFib) from cFib-immunized HLA-DR4 Tg mouse.** To normalize the variability, relative peak areas of $G1F^{2+}$ or $G2F^{2+}$ were deliberately set at 100%. Data for ratios of glycosylation are shown as mean ± standard deviation [*n*=5 for all types of IgG1]. Data were analyzed by one-way analysis of variance (ANOVA) with Tukey-Kramer *post hoc* test (*$P$ <0.05; **$P$ <0.01; ***$P$ <0.001).

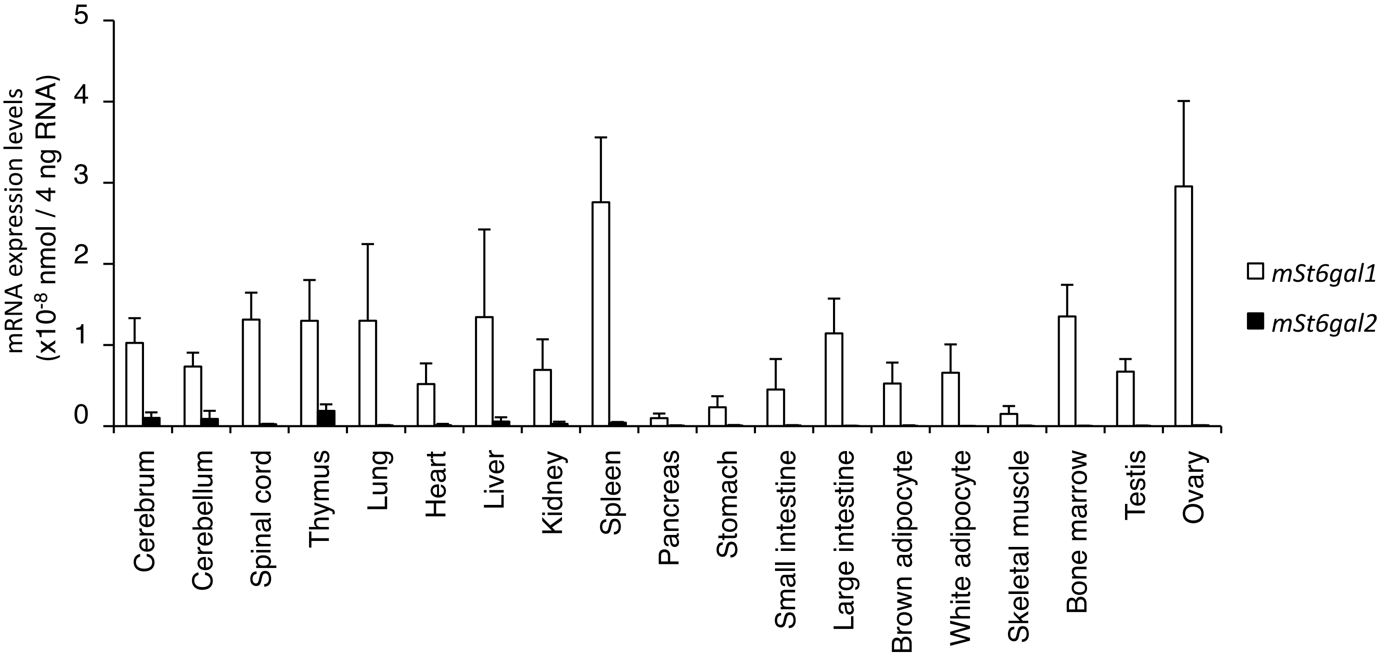


**Supplementary Figure 11. Expression levels of *mSt6gal1* and *mSt6gal2* genes in major tissues of B6 mice.** Expression levels of *mSt6gal1* and *mSt6gal2* genes were evaluated by real-time reverse transcription-polymerase chain reaction (RT-PCR) using *mSt6gal1* or *mSt6gal2* cDNA vectors as standard. Data are shown as mean ± standard deviation [*n*=4 for both genes].

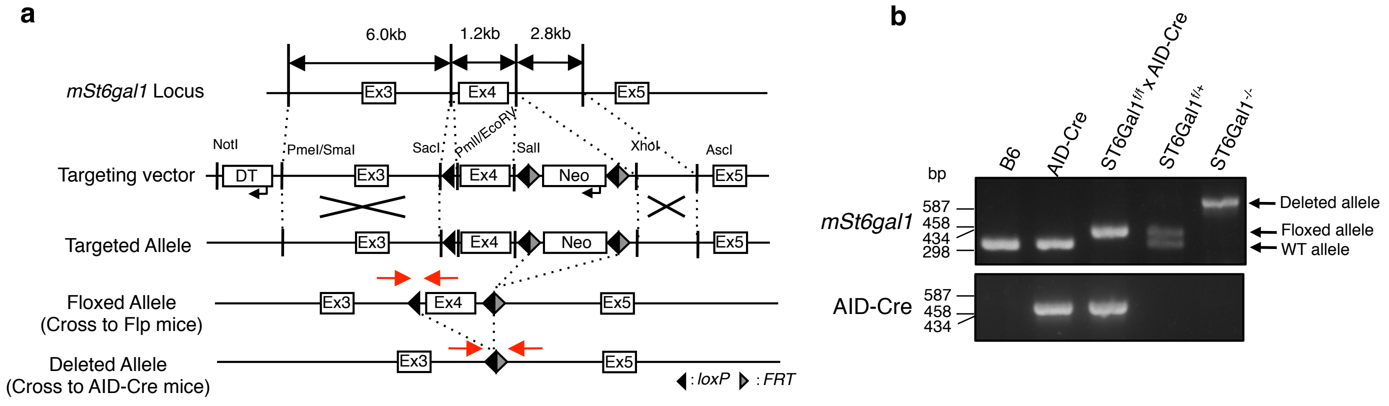


**Supplementary Figure 12. Gene targeting of *mSt6gal1* gene in activated B cell.** (**a**) A targeting vector included a floxed exon 4 encoding catalytic domain (L motif) of *mST6Gal1* and a floxed *Neo* cassette. The *Neo* cassette was also inserted between two *FRT* sites. Targeted allele-containing B6 mice were mated with Flp mice for deleting the *Neo* cassette, and subsequently crossed with AID-Cre mice for Cre-loxP recombination. Red arrows, genotyping primer sites. (**b**) PCR genotyping for the targeted *mSt6gal1* alleles and the AID-Cre-containing allele of B6, AID-Cre, ST6Gal1$^{f/f}$ AID-Cre, ST6Gal1$^{f/+}$ and ST6Gal1$^{-/-}$ (germline deletion) mice.

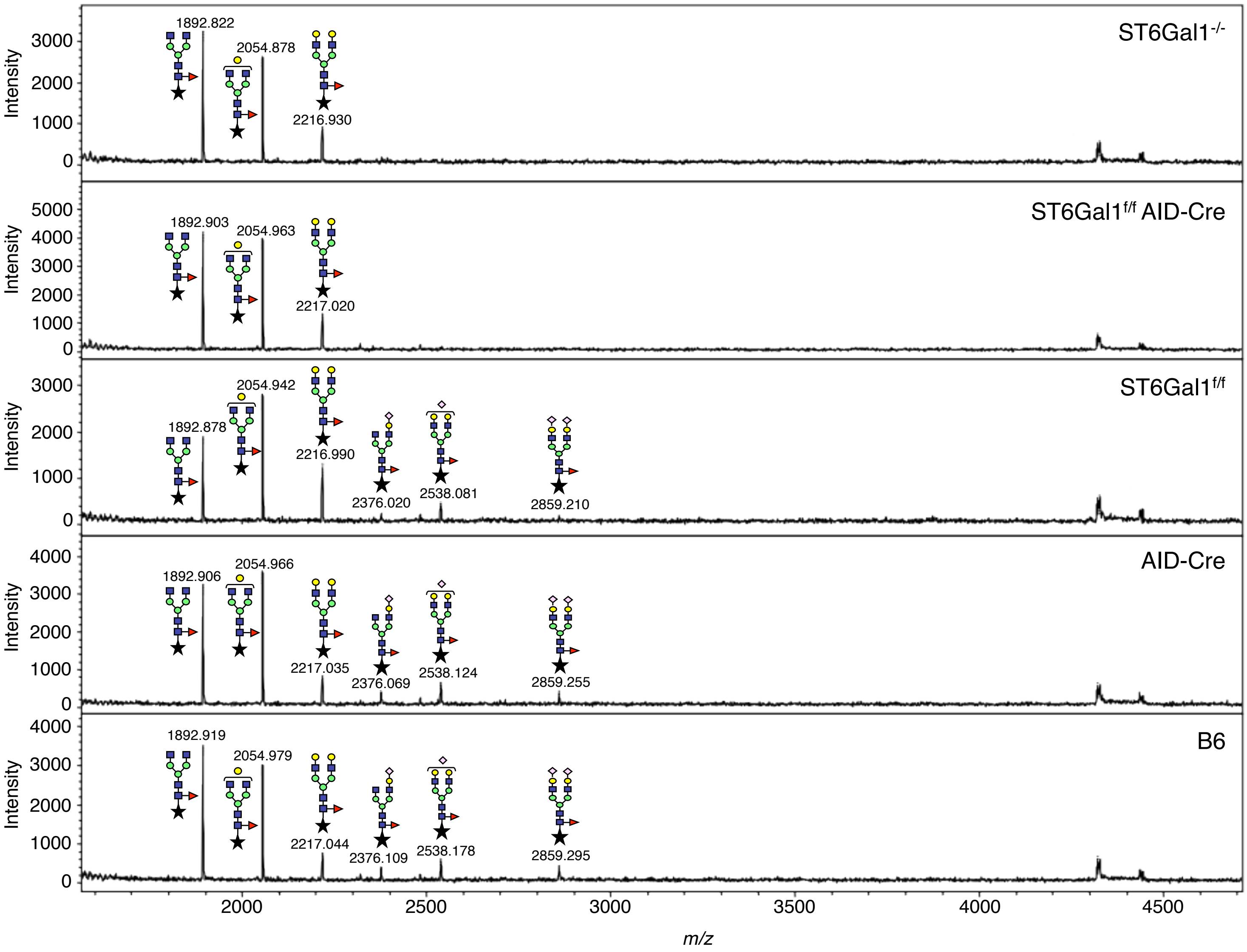


**Supplementary Figure 13. MALDI-TOF MS analysis of IgG1 Fc glycans from individual mice.** IgG1 Fc glycans in total IgG (10 μg) from naïve ST6Gal1$^{-/-}$ mice, and those in flow-through of Col II columns from CIA-induced B6, AID-Cre, ST6Gal1$^{f/f}$ AID-Cre or ST6Gal1$^{f/f}$ mice were analyzed by MALDI-TOF MS.

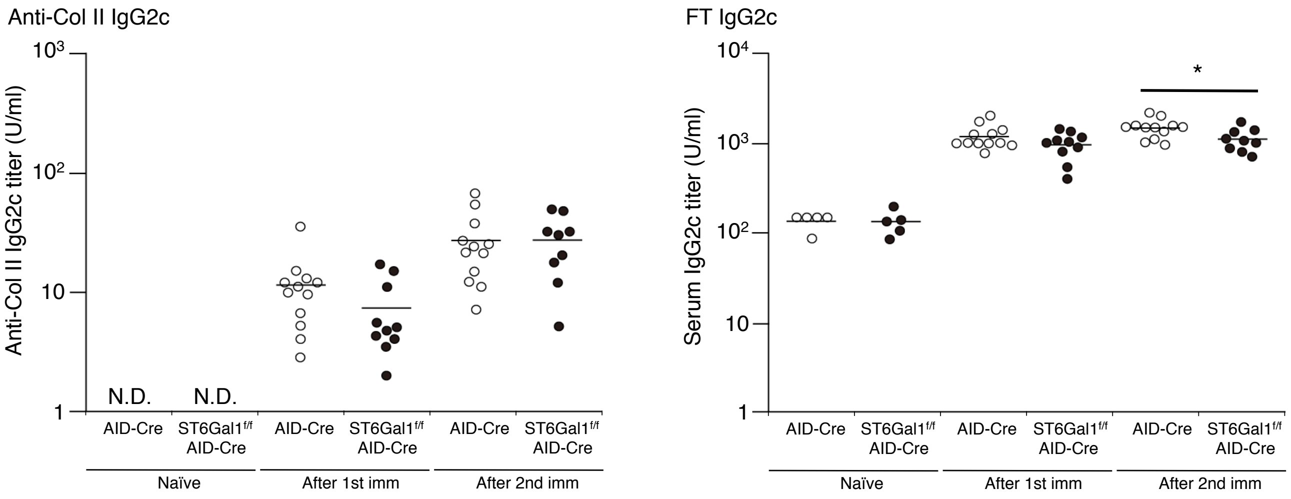


**Supplementary Figure 14. IgG2c levels in AID-Cre and ST6Gal1$^{f/f}$ AID-Cre mouse.** Anti-Col II IgG2c and total IgG2c titers in serum were determined by ELISA after 1$^{st}$ and 2$^{nd}$ immunization. Mean values were presented as bars. Each circle represents the result from an individual mouse. N.D., not detectable. The data are representative of three independent experiments. Data were analyzed by two-tailed Student's *t* test (**P* <0.05; ****P* <0.001).

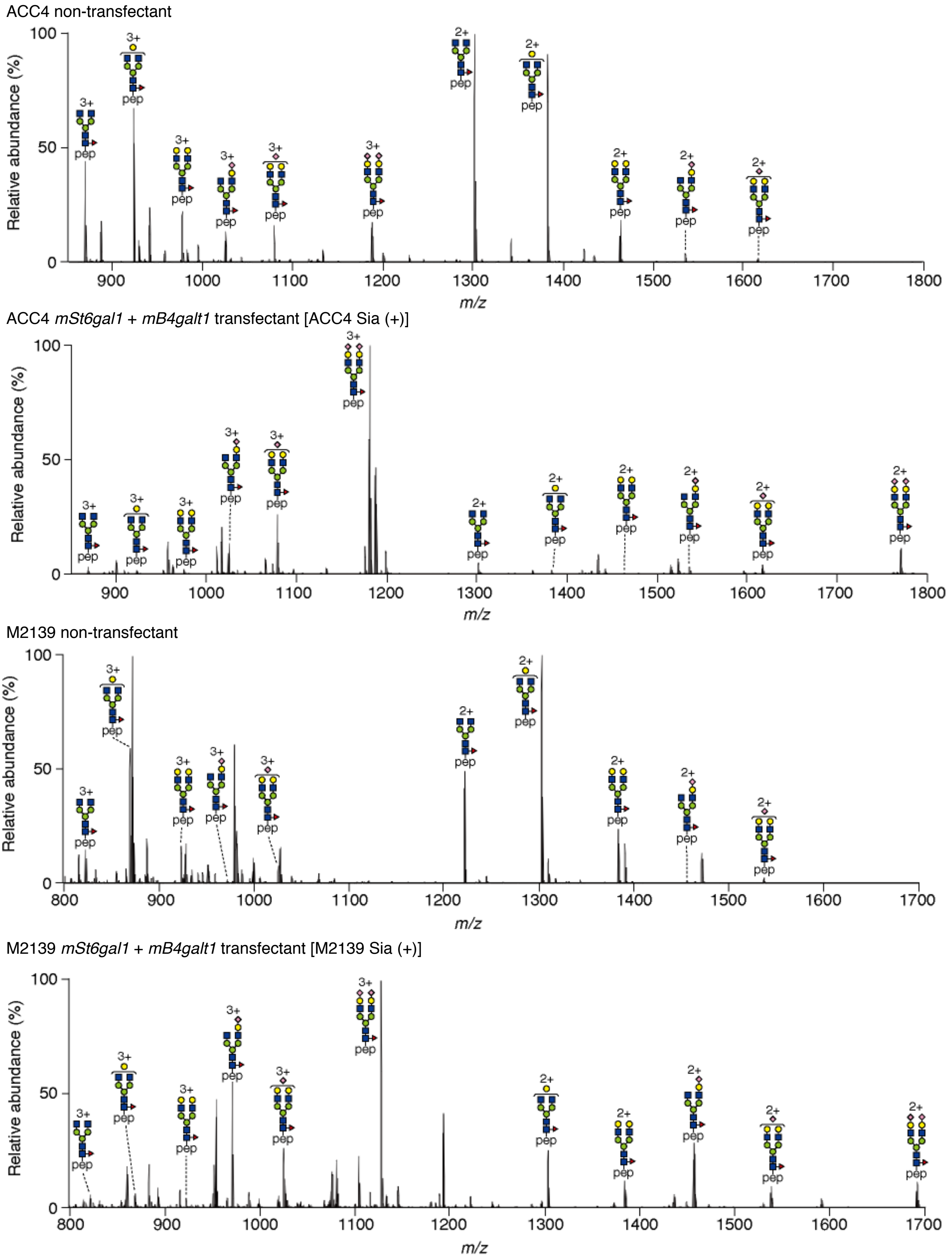


**Supplementary Figure 15. LC-ESI-MS analysis of IgG Fc glycans in ACC4 and M2139 with or without overexpression of *mSt6gal1* + *mB4galt1* cDNA.** Mass spectrometry profiles of ACC4 and M2139 (10 μg) are presented. The intensities of glycoforms with highest peak were deliberately set at 100%.

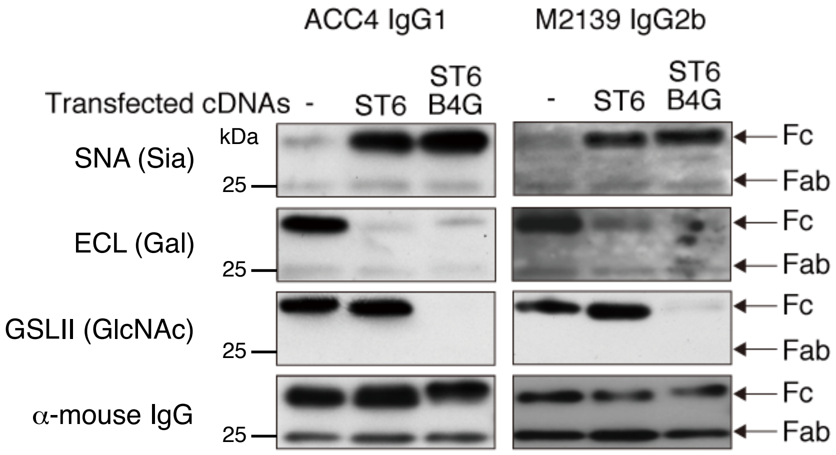


**Supplementary Figure 16. Glycoform analysis of ACC4 and M2139 by lectin blotting.** Lectin blotting analysis of papain-digested ACC4 (left panel) and M2139 (right panel) was performed to detect terminal α2,6 sialic acid [SNA (Sia)], galactose [ECL (Gal)], and *N*-acetylglucosamine [GSL II (GlcNAc)]. The amount of loaded IgG was evaluated by western blotting analysis using anti-mouse IgG. ST6, *mSt6gal1*; B4G, *mB4galt1*.

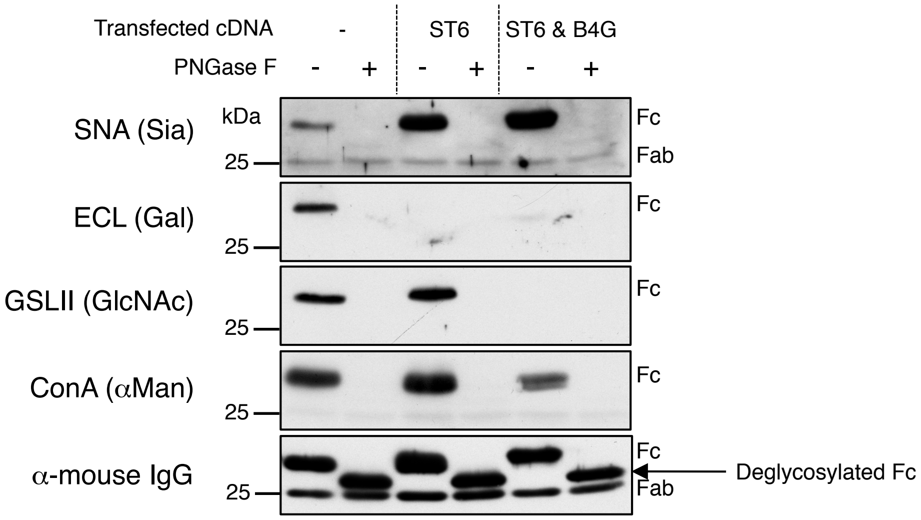


**Supplementary Figure 17. Altered lectin binding of ACC4 by PNGase F treatment.** Lectin blotting analysis of papain-digested ACC4 was performed after PNGase F treatment following SDS (1%) denaturation to detect terminal sialic acid [SNA (Sia)], galactose [ECL (Gal)], *N*-acetylglucosamine [GSL II (GlcNAc)], and α-linked Mannose [ConA (αMan)]. The amount of loaded IgG was evaluated by Western blotting analysis using anti-mouse IgG. ST6, *mSt6gal1*; B4G, *mB4galt1*.

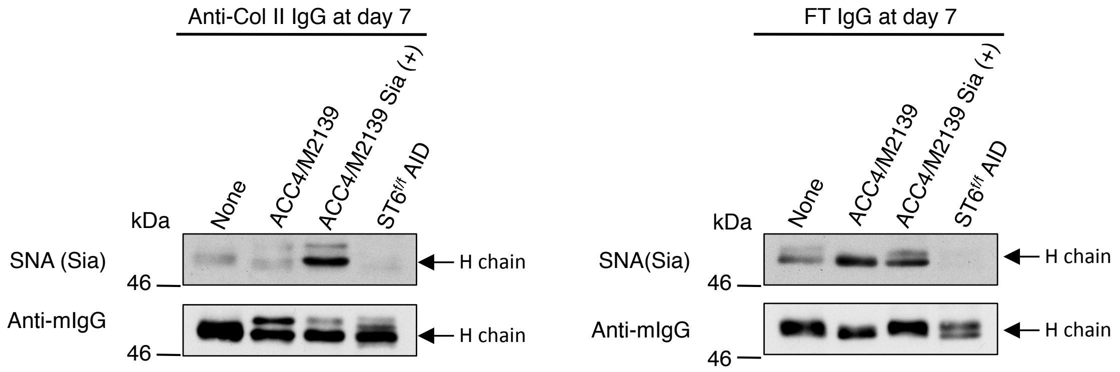


**Supplementary Figure 18. Sialylation pattern of anti-Col II IgG and flow through (FT) IgG from serum of CIA-induced DBA/1 mouse with or without the infusion of ACC4/M2139 or ACC4/M2139 Sia (+) antibodies.** Lectin blotting analysis of anti-Col II IgG and FT IgG in serum of CIA mouse injected with or without ACC4/M2139 or ACC4/M2139 Sia (+) antibodies was performed to detect terminal sialic acid [SNA (Sia)]. The amount of loaded IgG was evaluated by Western blotting analysis using anti-mouse IgG. Purification of anti-Col II IgG and FT IgG from serum of CIA-induced DBA/1 mice were performed as Supplementary Figure 5b.

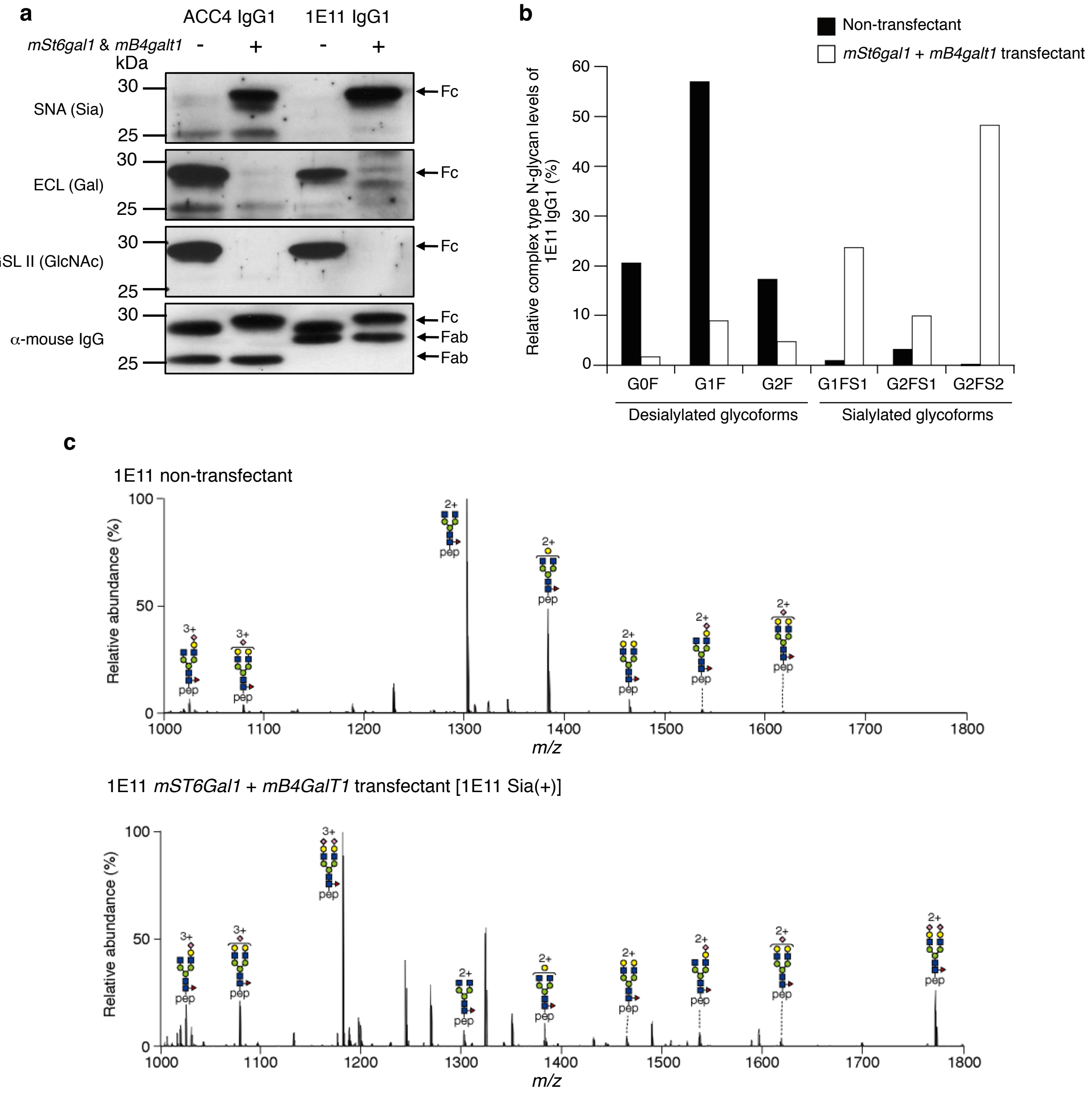


**Supplementary Figure 19. Sialylation of irrelevant IgG1, 1E11 with or without overexpression of *mSt6gal1* + *mB4galt1* cDNA.** (**a**) Lectin blotting analysis of the papain-digested 1E11 was performed to detect terminal sialic acid [SNA (Sia)], galactose [ECL (Gal)], and *N*-acetylglucosamine [GSL II (GlcNAc)]. The amount of loaded IgG was evaluated by Western blotting analysis using anti-mouse IgG. (**b**) LC-ESI-MS analysis of 1E11 was performed to detect desialylated glycoforms (G0F, G1F, and G2F) and sialylated glycoforms (G1FS1, G2FS1, and G2FS2) of IgG Fc glycans. To normalize the variability, summation of peak areas of all complex type N-glycans were deliberately set at 100%. (**c**) Mass spectrometry profiles of 1E11 (10 μg) are presented. The intensities of glycoforms with highest peak were deliberately set at 100%.

Figure2b

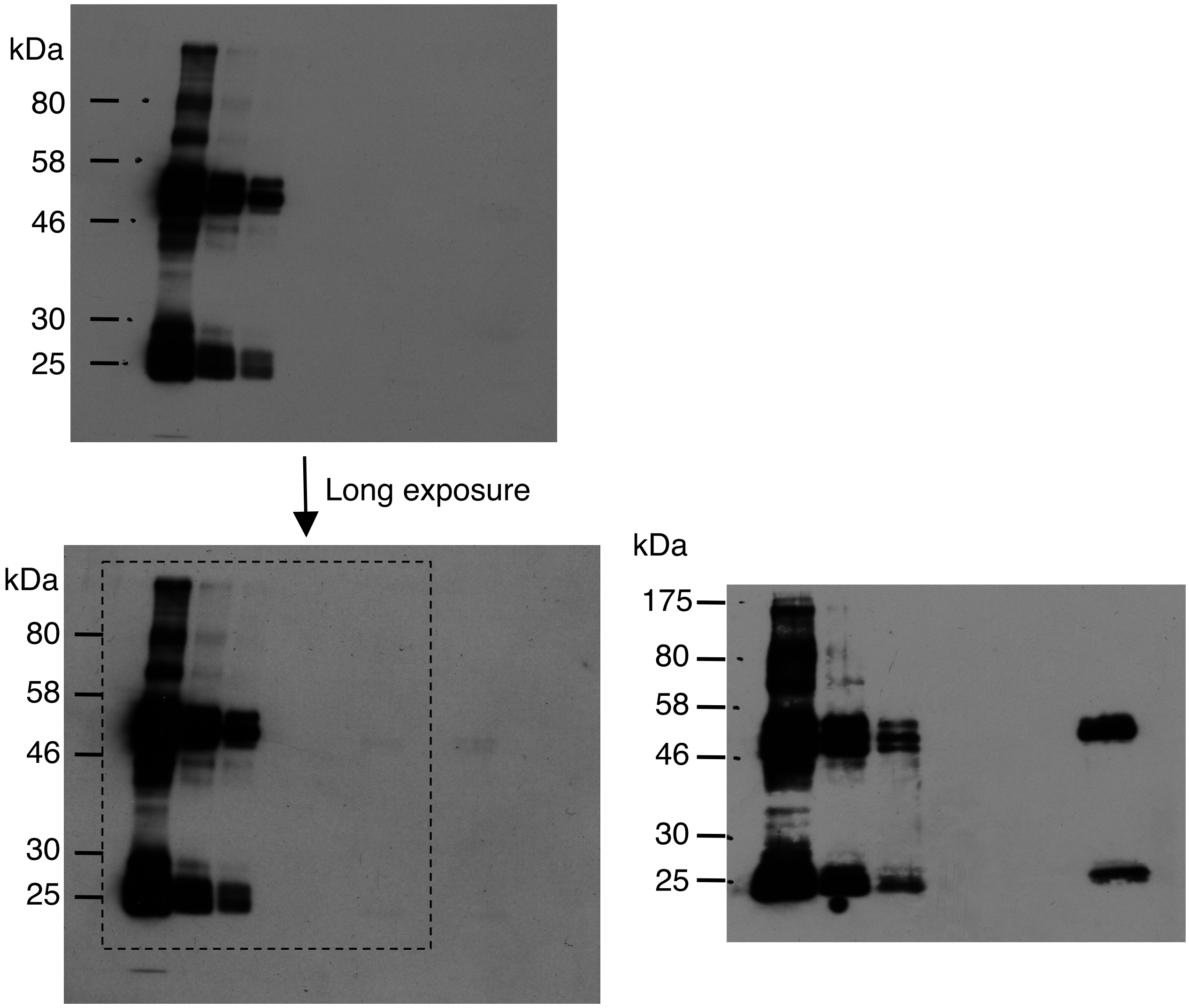


Figure3b

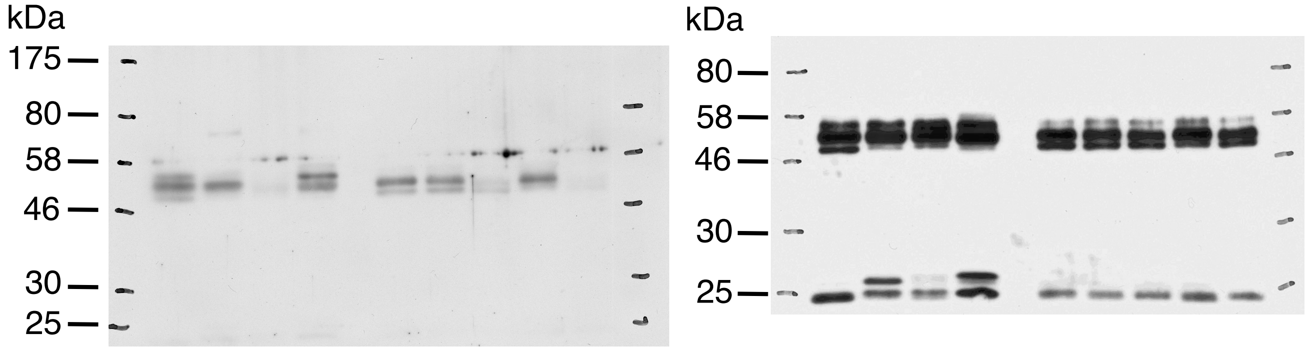


**Supplementary Figure 20.** Original images of the Western blotting analyses in the main figures.

**Supplementary Table 1.** Glycoforms of human IgG1 Fc by mass spectrometry in Figure 1c.

| No. | Glycoform | Compositions | 2+ *m/z* | 3+ *m/z* |
|---|---|---|---|---|
| 1 | G1FS1* | H4N4F1S1 | 1544.101 | 1029.736 |
| 2 | G2FS1 | H5N4F1S1 | 1625.127 | 1083.754 |
| 3 | G0F-GlcNAc-Man | H2N3F1 | 1134.960 | 756.976 |
| 4 | G2FS2 | H5N4F1S2 | 1770.675 | 1180.786 |
| 5 | G0F-GlcNAc | H3N3F1 | 1215.987 | 810.994 |
| 6 | G0 | H3N4 | 1244.498 | 830.001 |
| 7 | G1F-GlcNAc | H4N3F1 | 1297.013 | 865.011 |
| 8 | G0F | H3N4F1 | 1317.527 | 878.687 |
| 9 | G1 | H4N4 | 1325.524 | 884.018 |
| 10 | G0B | H3N5 | 1346.037 | 897.694 |
| 11 | G1F | H4N4F1 | 1398.553 | 932.704 |
| 12 | G2 | H5N4 | 1406.550 | 938.036 |
| 13 | G0BF | H3N5F1 | 1419.066 | 946.380 |
| 14 | G1B | H4N5 | 1427.064 | 951.712 |
| 15 | G2F | H5N4F1 | 1479.579 | 986.722 |
| 16 | G1BF | H4N5F1 | 1500.093 | 1000.398 |
| 17 | G2BF | H5N5F1 | 1581.119 | 1054.415 |

* A sialic acid (S) in purified IgG from human is composed of Neu5Ac.

**Supplementary Table 2.** Glycoforms of DBA/1 mouse IgG1 Fc by mass spectrometry in Figure 2c.

| No. | Glycoform | Compositions | 2+ *m/z* | 3+ *m/z* |
|---|---|---|---|---|
| 1 | G1FS1* | H4N4F1S1 | 1536.103 | 1024.405 |
| 2 | G2FS1 | H5N4F1S1 | 1617.130 | 1078.422 |
| 3 | G0F-GlcNAc-Man | H2N3F1 | 1118.966 | 746.313 |
| 4 | G2FS2 | H5N4F1S2 | 1770.675 | 1180.786 |
| 5 | G0F-GlcNAc | H3N3F1 | 1199.992 | 800.330 |
| 6 | G0 | H3N4 | 1228.503 | 819.338 |
| 7 | G1F-GlcNAc | H4N3F1 | 1281.018 | 854.348 |
| 8 | G0F | H3N4F1 | 1301.532 | 868.024 |
| 9 | G1F | H4N4F1 | 1382.558 | 922.041 |
| 10 | G2F | H5N4F1 | 1463.584 | 976.059 |

* A sialic acid (S) in purified IgG from mouse is composed of Neu5Gc.

**Supplementary methods**

**LC-ESI-MS analysis for IgG from RA patients and RA model mice (Figure 1 and 2)**

Aliquots of the collected IgG fractions (10 µg) were diluted to 50 µl with 20 mM ammonium acetate. The samples were supplemented with 200 µl of cold acetone, and kept at -20 °C overnight, then centrifuged at 10,000 x g for 10 min at 4 ◦C, and the supernatants were removed. The pellets were dissolved in 40 µl of 50% 1,1,1,3,3,3-Hexafluoro-2-propanol (Wako Pure Chemical Industries, Ltd.) containing 100 mM Tris-HCl, pH 8.0 and 5 mM EDTA. The solutions were added with 1 µl of 125 mM dithiothreitol (DTT), heated at 55°C for 30 min, and let cool to room temperature for 15 min. The solutions were added with 1.2 µl of 250 mM iodoacetic acid, and incubated at 30 °C for 30 min in dark. The solutions were supplemented with 190 µl of acetone - water- 2-isopropanol (10:3:2) containing 5 mM ammonium acetate and 0.5 mM DTT, vortexed for 10 sec, and centrifuged for 10 min at 10,000 x g at 25°C. The supernatants were removed, and the pellets were dissolved in 18 µl of 40 mM ammonium bicarbonate. Two µl of 0.1 µg/l modified trypsin solution was added, and samples were incubated at 37 °C for 5 hr. Tryptic digests were centrifuged at 10,000 x g for 1 min and 4 µl of aliquots were diluted to 25 µl with 0.1% trifluoroacetic acid (TFA).

Tryptic digests were analyzed by an LC-ESI-MS system consisting of a Paradigm MS4 HPLC system (Michrome Bioresources, Inc.) and Orbitrap Elite hybrid MS system

(Thermofisher Scientific, Inc.) equipped with a nanoESI ion source. Aliquots (0.1 µg of IgG) of the digests were loaded onto a L-column ODS trapping column (0.3 x 5 mm, Chemicals Evaluation and Research Institute), which were preconditioned with 0.1% TFA containing 2% acetonitrile, and desalted using 10 µl of 0.1% TFA containing 2% acetonitrile. Then, the trap column was inversely connected on-line in a series with the analytical column. Separation was achieved on a MonoCap C18 High Resolution 750（0.2 x 750 mm, GL Sciences Inc.）using a flow rate of 2 µl/min with the following gradient of mobile phase A (0.1% formic acid in 2% acetonitrile) and mobile phase B (0.1% formic acid in 90 % acetonitrile): 0 min 5 % B, 2 min 5 % B, 47 min 80 % B, 52 min 80 % B, 55 min 5 % B and 65 min 5 % B. The MS conditions were as follows: electrospray voltage of 2.4 kV in the positive ion mode; capillary temperature of 270°C; *m/z* range, 400-2000; resolution 15,000. Assignment of the detected glycopeptides was carried out on the basis of the deduced oligosaccharide compositions from mass spectrometry and knowledge of IgG N-glycosylation[1-3]. Peak area of IgG tryptic glycopeptide was obtained from extracted ion chromatogram of first isotopic peak.

**LC-ESI-MS analysis for IgG from RA patients and Healthy donors (Supplementary figure 5)**

Thirty µl of IgG fractions (5 µg) were added with 10 µl of 500 mM ammonium hydrogen carbonate and 2 µl of 500 mM dithiothreitol (DTT), heated at 60°C for 45 min, and let cool to room temperature for 15 min. The solutions were added with 2 µl of 500 mM iodoacetamide, and incubated for 30 min in dark. The solutions were supplemented with 1 µl of 500 mM DTT, vortexed for 10 sec, and added water to make 50 µl. Zero point five µl of trypsin solution (0.5 µg/µl) was added, and samples were incubated at 37 °C for over-night. The solutions were supplemented with 250 µl of cold acetone, vortexed for 10 sec, and samples were incubated at -25 °C for over-night. Tryptic digests were centrifuged at 12,000 x g at 20°C for 10 min. The supernatants were removed, and the pellets were dissolved in 12 µl of 0.1% formic acid. The digests were separated on a NANO HPLC CAPILLARY COLUMN, 75µm ID x 120mm, 3µm, C18 (Nikkyo Technos) at a flow rate of 300 nl/min in an LC-ESI-MS system consisting of an EASY-nLC 1000 (Thermo Scientific). The mobile phases were 0.1 % formic acid (A buffer) and 0.1 % formic acid in acetonitrile (B buffer). The digests were eluted with a linear gradient of 0–32% of B buffer for 40 min followed by 32-100% of B buffer for 2 min. Mass spectrometric analysis was performed using Q Exactive mass spectrometer (Thermofisher Scientific, Inc.) equipped with Nanospray Flex Ion Source (Thermo Scientific). The electrospray voltage was 2.0 kV, the resolution was 70,000, and the scan range was m/z 700–2,000.

**LC-ESI-MS analysis for IgG from ACC4, M2139 and 1E11 hybridomas**

Tryptic digests of IgG1 form ACC4 or ACC4 Sia (+), 1E11 or 1E11 Sia (+), and IgG2 from M2139 or M2139 Sia (+) were separated on a NANO HPLC CAPILLARY COLUMN, 75µm ID x 120mm, 3µm, C18 (Nikkyo Technos) at a flow rate of 300 nl/min in an LC-ESI-MS system consisting of an EASY-nLC 1000 (Thermo Scientific). The mobile phases were 0.1 % formic acid containing 2% acetonitrile (A buffer) and 0.1 % formic acid containing 90% acetonitrile (B buffer). The digests were eluted with a linear gradient of 0–4% of B buffer for 1 min followed by 4–32% of B buffer for 59 min. Mass spectrometric analysis was performed using Orbitrap Fusion Tribrid mass spectrometer (Thermofisher Scientific, Inc.) equipped with Nanospray Flex Ion Source (Thermo Scientific). The electrospray voltage was 2.0 kV, the resolution was 120,000, and the scan range was m/z 350–2,000.

**MALDI-TOF MS analysis**

Ten µg of purified IgGs from CIA-induced ST6Gal1$^{f/f}$ AID-Cre, ST6Gal1$^{f/f}$, AID-Cre and C57BL/6j mice and total serum IgG from ST6Gal1 KO mice were dissolved in 100 mM ammonium bicarbonate buffer. The solutions (60 µl) were reduced by dithiothreitol (DTT)

for 30 min at 60 °C followed by alkylation with 10 μl of 123 mM iodoacetamide (IAA) by incubation in the dark at room temperature for 60 min. The mixture was then treated with 1 μl of 40 units/μl trypsin for 60 min at 37 °C, followed by heat inactivation of the enzyme for 10 min at 90 °C. After being cooled to room temperature, N-glycans were enzymatically released from trypsin-digested glycol- peptides by incubation with 1 unit of PNGase F (Roche Applied Science, Basel, Switzerland) overnight at 37 °C. PNGase F-treated samples were dropped onto BlotGlyco beads (Sumitomo Bakelite Co., Tokyo, Japan) in a filter plate, and followed by addition of 2% acetic acid/acetonitrile. After incubation for 60 min at 80 °C, to covalently ligate glycans to beads, the beads were sequentially washed with 2 M guanidine hydrochloride, distilled water, and 1% triethylamine/methanol to remove nonspecifically bound impurities. After washing, the beads were treated with 10% acetic anhydride/methanol for 30 min at ambient temperature to quench the hydrazide groups. The beads were then washed serially with 10 mM hydrochloric acid, distilled water, methanol, and dimethyl sulfoxide (DMSO). On-bead methyl esterification was performed by the reaction with 500 mM 1-methyl-3-p-tolyltriazene/DMSO solution for 60 min at 60 °C. After the beads were washed with methanol and distilled water, the immobilized glycans were finally released and recovered as N-α-((aminooxy)acetyl) -tryptophanylarginine methyl ester (aoWR) derivatives by adding 20 mM aoWR and 2% acetic acid/acetonitrile

and incubated for 1 hour at 80 °C. The resulting aoWR-labeled glycans were recovered by washing the beads with distilled water, and the collected solution was further purified with a solid-phase extraction column (Cleanup column, Sumitomo Bakelite Co., Ltd., Tokyo, Japan) to remove the excess aoWR. The purified solution was mixed with 2,5-dihydroxybenzoic acid solution and subsequently subjected to MALDI-TOF analysis. All measurements were performed with an Autoflex III TOF/TOF mass spectrometer equipped with a reflector and controlled by the FlexControl 3.0 software package (Bruker Daltonics GmbH, Bremen, Germany). The peaks were detected generally as a formula of [M + H]+ ions. In MALDI-TOF MS reflector mode, ions generated by a Smartbeam (pulsed UV nitrogen laser, $\lambda$ = 337 nm, 5 Hz) were accelerated to a kinetic energy of 23.5 kV. Masses were automatically annotated by using FlexAnalysis 2.0. External calibration of MALDI mass spectra was carried out using singly charged monoisotopic peaks of a mixture of human angiotensin II (m/z 1046.542), bombesin (m/z 1619.823), adrenocorticotropin (18–39) (m/z 2465.199), and somatostatin 28 (m/z 3147.472). All measurements were performed as follows: 1 ml of the sample solution was mixed with 1 ml of 2,5-dihydroxybenzoic acid (10 mg/ml in 30% ACN), and 1 ml of the resulting mixture was subjected to MALDI-TOF mass analysis. Structural identification of glycans was performed using of a database for glycan structures (http://web.expasy.org/glycomod/).

**Supplementary References**

1. Parekh, R.B., *et al.* Association of rheumatoid arthritis and primary osteoarthritis with changes in the glycosylation pattern of total serum IgG. *Nature* **316**, 452-457 (1985).

2. Wuhrer, M., *et al.* Glycosylation profiling of immunoglobulin G (IgG) subclasses from human serum. *Proteomics* **7**, 4070-4081 (2007).

3. Harazono, A., *et al.* Simultaneous glycosylation analysis of human serum glycoproteins by high-performance liquid chromatography/tandem mass spectrometry. *J. Chromatogr. B Analyt. Technol. Biomed. Life Sci.* **869**, 20-30 (2008).
